# Molecularly Imprinted Polyaniline-Coated Cu-Zeolitic Imidazolate Framework Nanoparticles: Uricase-Mimicking “Polynanozyme” Catalyzing Uric Acid Oxidation

**DOI:** 10.1021/acsnano.4c16272

**Published:** 2025-03-05

**Authors:** Xinghua Chen, Yi Wu, Yunlong Qin, Raanan Carmieli, Inna Popov, Vitaly Gutkin, Chunhai Fan, Itamar Willner

**Affiliations:** † Institute of Chemistry, The Hebrew University of Jerusalem, Jerusalem 91904, Israel; ‡ Department of Chemical Research Support, Weizmann Institute of Science, Rehovot 76100, Israel; § The Center for Nanoscience and Nanotechnology, The Hebrew University of Jerusalem, Jerusalem 91904, Israel; ∥ School of Chemistry and Chemical Engineering, Nanjing University of Science and Technology, Nanjing 210094, China; ⊥ School of Chemistry and Chemical Engineering, Frontiers Science Center for Transformative Molecules, National Center for Translational Medicine, Shanghai Jiao Tong University, Shanghai 200240, China

**Keywords:** nanozyme, superoxide radical anion, nanoparticle catalysis, sensor, artificial enzyme

## Abstract

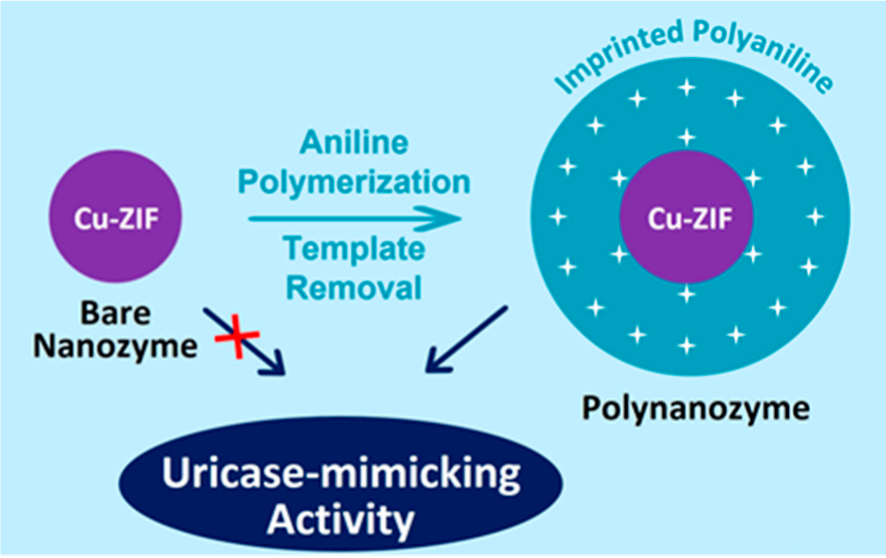

One of the drawbacks of nanozyme catalytic functions rests in their moderate catalytic activities due to the lack of effective binding sites concentrating the reaction substrate at the nanozyme catalytic interface. Methods to concentrate the substrates at the catalytic interface are essential to improving nanozyme functions. The present study addresses this goal by designing uric acid (UA) molecular-imprinted polyaniline (PAn)-coated Cu-zeolitic imidazolate framework (Cu-ZIF) nanoparticles as superior nanozymes, “polynanozymes”, catalyzing the H_2_O_2_ oxidation of UA to allantoin (peroxidase activity) or the aerobic, uricase mimicking, oxidation of UA to allantoin (oxidase activity). While bare Cu-ZIF nanoparticles reveal only peroxidase activity and the nonimprinted PAn-coated Cu-ZIF nanoparticles reveal inhibited peroxidase activity, the molecular-imprinted PAn-coated Cu-ZIF nanoparticles reveal a 6.1-fold enhanced peroxidase activity, attributed to the concentration of the UA substrate at the catalytic nanoparticle interface. Moreover, the catalytic aerobic oxidation of UA to allantoin by the imprinted PAn-coated Cu-ZIF nanoparticles is lacking in the bare particles, demonstrating the evolved catalytic functions in the molecularly imprinted polynanozymes. Mechanistic characterization of the system reveals that within the UA molecular imprinting process of the PAn coating, Cu^+^ reactive units are generated within the Cu-ZIF nanoparticles, and these provide reactive sites for generating O_2_^–•^ as an intermediate agent guiding the oxidase activities of the nanoparticles. The study highlights the practical utility of molecular-imprinted polynanozymes in catalytic pathways lacking in the bare nanozymes, thus broadening the scope of nanozyme systems.

## Introduction

Substantial recent research efforts are directed to the development of nanoparticle-based catalysts, nanozymes.^1−3^ Diverse inorganic nanoparticles, such as metal nanoparticles, such as Pt,^4,5^ Pd,^6^ or Au^7^ nanoparticles, metal oxide nanoparticles, such as CeO_2_,^8,9^ MoO_3_,^10^ or V_2_O_5_,^11^ carbon-based materials,^12,13^ such as C-dots, graphene oxide, and composite nanoparticles or clusters, e.g., C_3_N_4_,^14^ or Prussian blue^15,16^ were reported to catalyze different chemical transformations. Also, organic nanoparticles composed of polydopamine^17^ or melanin^18^ were reported to reveal catalytic properties. Moreover, metal–organic framework nanoparticles, NMOFs, provide versatile catalytic nanozyme frameworks,^19^ where the ions linked to the ligands composing the nanoparticles,^20,21^ metal ions anchored to the cross-linking ligands^22^ or catalytic particles,^23^ or enzymes^24^ incorporated in the porous structure of NMOFs yield the catalytic nanozyme functions of the frameworks. Diverse chemical transformations were catalyzed by nanozymes including peroxidase,^25,26^ oxidase,^27,28^ catalase,^29^ superoxide dismutase,^30^ hydrolase,^31^ and isomerase^32^ processes emulating native enzymes. Different applications of nanozymes were reported, including their use for sensing,^33^ electrochemical sensing,^34^ imaging and therapeutic agents for various diseases including cancer,^35,36^ Parkinson’s^36^ and Alzheimer’s^37^ diseases, and wound healing.^38^ Also, nanozymes were applied to antibacterial agents^39,40^ and catalysts for degradation of environmental pollutants and wastes.^41,42^ In addition, chemically modified nanozymes were used as bioreactor systems driving catalytic cascades.^43^

While substantial progress in the development of nanozymes and their applications have been demonstrated, the systems are not free of limitations: (i) their catalytic activities and catalytic turnovers are still low as compared to native enzymes. This may be attributed to the lack of binding sites, concentrating the reactive substrate in the spatial proximity of the catalytic sites (“molarity effect”). (ii) The selectivity of the nanozymes toward specific substrates, particularly stereoselectivity and enantioselectivity, is scarce. Again, the lack of selectivity might be attributed to the lack of specific binding sites or chiral binding sites for the substrate. Different methods to overcome these limitations were reported including the functionalization of nanozymes, e.g., Cu^2+^-modified C-dots^44^ or Au nanoparticles,^45^ with nucleic acid aptamer strands acting as sequence-specific substrate binding sites, “aptananozymes”, and the covalent modification of Cu^2+^-ion-functionalized C-dots with β-cyclodextrin receptor units as supramolecular nanozymes for the enhanced oxidation of dopamine.^14^ Also, the integration of nanozymes in molecularly imprinted polymers was suggested as a means to concentrate the substrate in spatial proximity to the catalytic sites, thereby enhancing the catalytic activity and selectivity of the nanozymes.^46^ Two general strategies to imprint molecular recognition sites in polymer matrices were reported.^47,48^ By one method, polymerizable monomers containing functional groups complementary to ligand functionalities associated with the template substrate (i.e., H-bonds, electrostatic interactions, or donor–acceptor complexes) were polymerized in the presence of the guest substrate, yielding after polymerization and washing-off of the guest substrate, molecular contours revealing relative binding affinities for the imprinted substrate. By a second approach, polymerizable monomers form supramolecular complexes with the guest substrate (e.g., boronate complexes). Polymerization of the supramolecular structures, followed by chemical cleavage of the bridging bonds, led to the size of functionally confined sites for binding of the guest substrate. Selective and chiroselective imprinted polymer frameworks for the association of the imprinted substrate were demonstrated, and diverse applications of the imprinted matrices for separation,^49,50^ catalysis,^51,52^ sensing,^53,54^ and controlled release^55,56^ applications were reported. While diverse chemical transformations of nanozymes were demonstrated, broadening the scope of chemical reactions driven by nanozymes and their applications is essential. Moreover, the chemical modification of nanozymes into a hybrid structure revealing catalytic functions absent in the bare nanoparticles is challenging.

Within the diverse NMOFs, the metal-ion imidazolate ligand bridged zeolitic imidazolate framework (ZIF) family is an important subclass of NMOFs.^57^ The assembly of the ZIF NMOFs in an aqueous system at ambient temperature is particularly important, as integration and protection of temperature-sensitive biomaterials, such as enzymes, antibodies, or nucleic acids, in these frameworks is feasible while retaining their biological functions. Moreover, different metal ions such as Zn^2+^, Co^2+^, Cu^2+^, and more were employed to cross-link the imidazolate ligand,^58,59^ and diverse metal ions used to dope the basic Zn^2+^-cross-linked ZIF-8 framework^60,61^ were reported to yield different catalytic ZIF frameworks. Also, the use of chemically modified imidazolate ligands with functional carboxaldehyde,^62^ amine,^63^ and more^64^ enhanced the secondary functionalization of the ZIF NMOFs, yielding hybrid structures exhibiting broad catalytic activities and versatile applications. The highly porous structure of ZIF NMOFs enabled the integration of molecular agents, nanoparticles, or proteins in the ZIF NMOF frameworks,^65^ and the hybrid structures were used for sensing,^66^ drug delivery,^67^ and NMOF-guided catalytic transformations.^24,68^ Also, chemical modification of the imidazolate composing the ZIF frameworks enabled the engineering of ZIF frameworks acting as bioreactors driving catalytic cascades of enhanced complexities.^69^ Recently, Co^2+^-ZIF-67 was reported^70^ as a peroxidase-mimicking nanozyme catalyzing the H_2_O_2_ oxidation of dopamine to aminochrome, the oxidation of NADH to NAD^+^, and the catalyzed generation of chemiluminescence in the presence of luminol/H_2_O_2_. Also, Co^2+^-ZIF-67 catalyzed the oxidation of aniline to polyaniline, and molecularly imprinted polyaniline-coated Co^2+^-ZIF-67 NMOFs demonstrated chiroselective oxidation of l/d-DOPA in the presence of H_2_O_2_.^70^

The catalyzed oxidation of uric acid, UA, to allantoin attracts substantial research efforts due to the therapeutic significance of the process. Gout is a common inflammatory arthritis disease reflected by the deposition of monosodium urate in peripheral joints and tissues.^71,72^ Inflammatory arthritis originates from hyperuricemia, uricase deficiency catalyzing the aerobic oxidation of uric acid to allantoin.^73^ The current treatment of gout involves the use of anti-inflammatory drugs and uricase-substituting agents.^74^ Not surprisingly, substantial efforts are directed to the development of uric acid-sensing platforms^75^ and catalytic agents to degrade uric acid deposits^76^ and to understand the basic mechanistic features of native uricase.^77^ Indeed, recent studies have reported the application of nanozymes as sensing and therapeutic materials for gout control.^78,79^

Here we report on the synthesis of a Cu-ZIF nanoparticle/nanofiber composite as a nanozyme catalyzing the H_2_O_2_ oxidation of UA to allantoin. Moreover, we find that Cu-ZIF NMOFs catalyze the H_2_O_2_ oxidation of aniline to polyaniline, PAn. This allowed the catalytic coating of the Cu-ZIF NMOFs with a film of PAn. The PAn-coated Cu-ZIF NMOFs demonstrate low catalytic activity toward the oxidation of UA as compared to the bare Cu-ZIF NMOFs. The molecular imprinting of the PAn coating with UA-imprinted sites yields, however, a highly efficient UA-imprinted PAn-coated Cu-ZIF nanozyme (“polynanozyme”) catalyzing the H_2_O_2_ oxidation of UA to allantoin. The UA-imprinted PAn-coated Cu-ZIF polynanozyme reveals substantially enhanced catalytic properties, as compared to the bare Cu-ZIF NMOFs. The enhanced catalytic activities are attributed to the concentration of UA at the imprinted sites (“molarity effect”), in the proximity of the NMOF catalytic sites. Moreover, we find that the imprinted PAn-coated Cu-ZIF polynanozyme reveals uricase-mimicking activities, catalyzing the aerobic oxidation of UA to allantoin. While the bare Cu-ZIF NMOFs lack uricase mimicking activities, the imprinted PAn-coated Cu-ZIF NMOFs reveal effective UA oxidase activities, demonstrating new catalytic functions of the “polynanozyme”, which are absent in the bare NMOF nanozyme. Besides introducing Cu-ZIF NMOFs as nanozymes catalyzing the oxidation of UA, the importance of the present study is reflected by demonstrating that molecular imprinting of polymer-coated NMOFs yields hybrid NMOFs, revealing effective catalytic activities absent in the bare NMOFs.

## Results and Discussion

### Synthesis, Characterizations, and Catalytic Peroxidase Functions of the Cu-ZIF NMOFs

**Figure 1:**
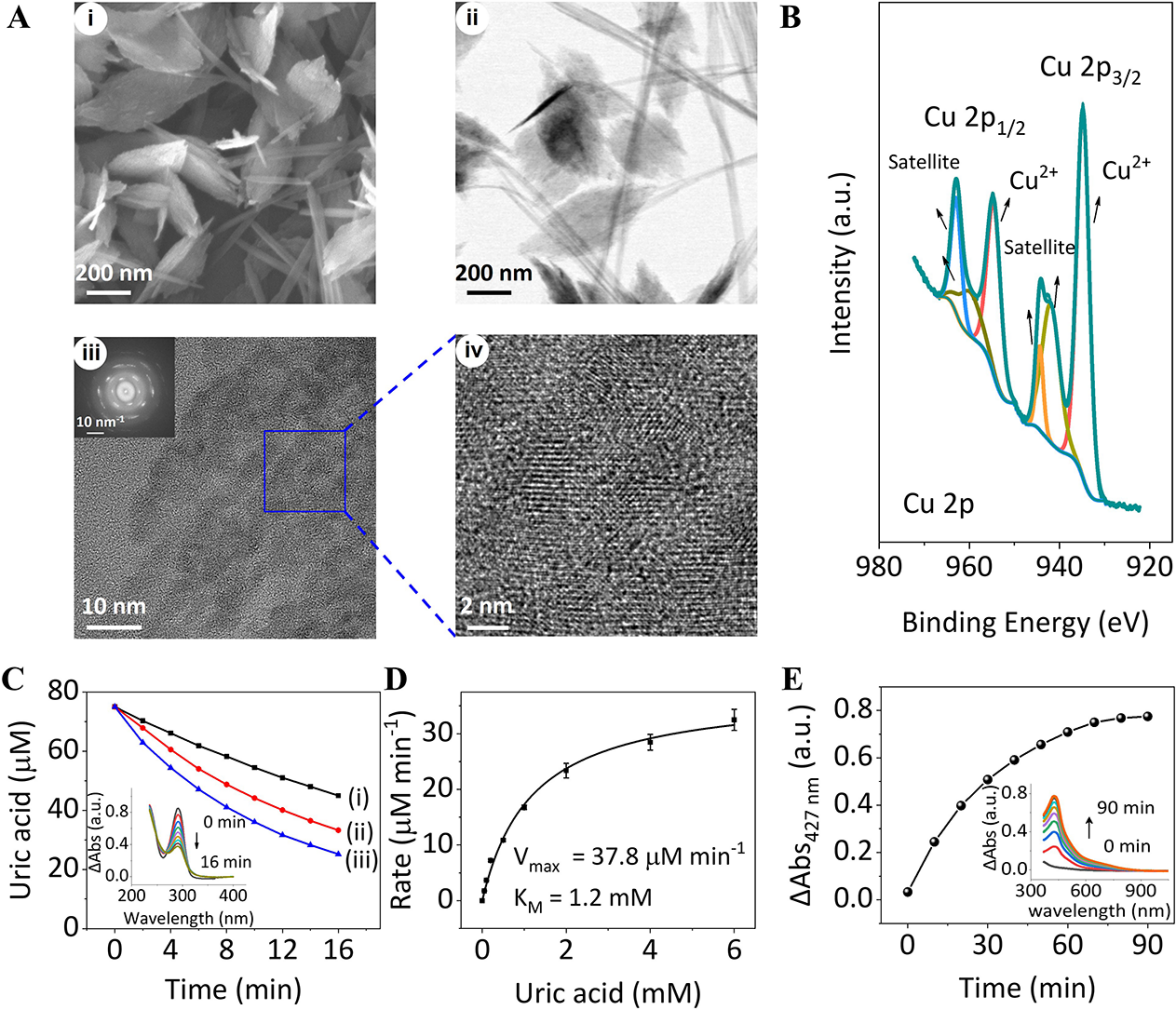
Structure, composition, and catalytic functions of Cu-ZIF NMOFs. (A) SEM image (i), TEM image (ii), HR-TEM image (iii), FFT image (10 nm^–1^); inset, (iii), and enlarged domain of the HR-TEM section (iv) of Cu-ZIF NMOFs. (B) Deconvoluted Cu 2p XPS spectra of Cu-ZIF NMOFs. (C) Time-dependent concentration changes of UA upon the catalyzed oxidation of UA by H_2_O_2_ to allantoin in the presence of different concentrations of Cu-ZIF NMOFs: (i) 25 μg mL^–1^; (ii) 50 μg mL^–1^; (iii) 75 μg mL^–1^. In all experiments, UA, 75 μM, and H_2_O_2_, 5 mM, were used. Inset, time-dependent absorbance changes of UA upon the Cu-ZIF NMOFs catalyzed oxidation of UA by H_2_O_2_: Cu-ZIF NMOFs, 50 μg mL^–1^; UA, 75 μM; H_2_O_2_, 5 mM. (D) Rates of UA oxidation by H_2_O_2_, 5 mM, in the presence of Cu-ZIF NMOFs, 50 μg mL^–1^, and variable concentrations of UA. (E) Time-dependent absorbance changes upon the Cu-ZIF NMOFs, 100 μg mL^–1^, catalyzed oxidation of aniline, 1.2 mM, by H_2_O_2_, 2 mM, to form polyaniline, PAn. Inset: time-dependent spectra of PAn upon oxidation of aniline, 1.2 mM, in the presence of H_2_O_2_, 2 mM, using Cu-ZIF NMOFs, 100 μg mL^–1^.

The Cu-ZIF NMOFs were prepared by mixing CuCl_2_ with 2-methylimidazole in the presence of hexadecyltrimethylammonium bromide at room temperature following the reported procedure.^58^ The NMOFs were analyzed by different spectroscopic and microscopic methods. Figure 1A depicts the scanning electron microscopy (SEM) image, Panel (i), and the transmission electron microscopy (TEM) image, Panel (ii), of the Cu-ZIF NMOFs. The images reveal that the NMOFs appear as a mixture of flakes and fibers, consistent with previous reports. The flakes reveal an “egg”-shaped structure with a length of 522 ± 260 nm and a middle width of 244 ± 88 nm, and the fibers reveal a length of 2 μm or longer (Figure S1). (Moreover, the mixture of flakes and fibers can be transformed into pure flake composition, without changing the functionalities of the flakes by cyclic treatment of the mixture with acetone/water; see Figure S2 and accompanying discussion.) The high-resolution TEM (HR-TEM) images are displayed in Panels (iii and iv). The accompanying fast Fourier transformation (FFT) image of the full area of Panel (iii) is displayed in the Panel (iii) inset. The powder X-ray diffraction (PXRD) spectrum corresponding to the NMOFs is displayed in Figure S3 (the detailed diffraction peaks and corresponding *d*-spacing are provided in Table S1). The Brunauer–Emmett–Teller (BET) specific surface area of the Cu-ZIF NMOFs corresponds to 486.4 m^2^ g^–1^, and the pore-size distribution is in the range of 1.7–5.5 nm (Figure S4). Moreover, Figure 1B depicts the deconvoluted Cu 2p X-ray photoelectron spectroscopy (XPS) spectra of the NMOFs, demonstrating that the Cu^2+^-ionic state is present in the Cu-ZIF NMOFs (for the XPS spectra of the other elements composing the NMOFs, see Figure S5).

Figure 1C depicts the time-dependent concentration changes upon oxidation of UA to allantoin by H_2_O_2_ in the presence of variable concentrations of the Cu-ZIF NMOFs. As the concentration of the NOMFs increases, the oxidation process is enhanced. Figure 1D shows the rates of UA oxidation in the presence of Cu-ZIF NMOFs, 50 μg mL^–1^, and H_2_O_2_, 5 mM, using variable concentrations of UA. As the concentration of the substrate increases, the rate of UA oxidation is higher and it reaches a saturation level at the UA concentration of ca. 6 mM. From this curve, the derived *K*_M_ and *V*_max_ values corresponding to 1.2 mM and 37.8 mM min^–1^ were derived. Moreover, we found that the Cu-ZIF NMOFs acted as nanozymes catalyzing the H_2_O_2_ oxidation of aniline to polyaniline, PAn, eq 1.^80^Figure 1E, inset, shows the temporal absorbance spectra of PAn in the solution, and Figure 1E depicts the temporal absorbance changes of PAn in solution (λ = 427 nm) upon formation of PAn. The results demonstrate a saturation kinetic, and under the specific experimental conditions after a time interval of ca. 80 min, the catalyzed polymerization process is blocked. Blocking of the polymerization process is attributed to the concomitant coating of the Cu-ZIF NMOFs, a process that blocks the catalytic sites on the nanoparticles toward oxidation of aniline in solution. The coating of the Cu-ZIF by PAn is visually observed by particles turning from light brown to dark brown. The coating of the Cu-ZIF NMOFs with the PAn provided, however, the basis for the study to be described below. (For characterization of the PAn-coated NMOFs, vide infra.)
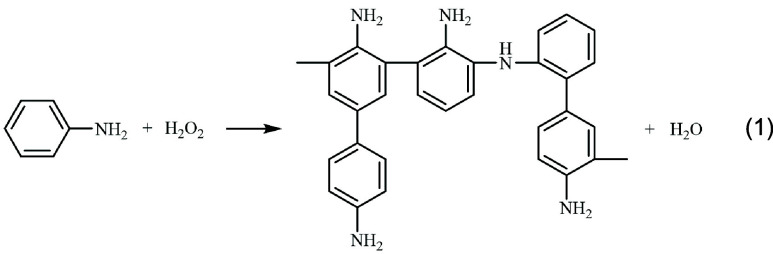
1

### Uric Acid Peroxidase/Oxidase Catalytic Functions of the Imprinted/Nonimprinted PAn-Coated Cu-ZIF NMOFs vs the Bare Cu-ZIF NMOFs

**Figure 2:**
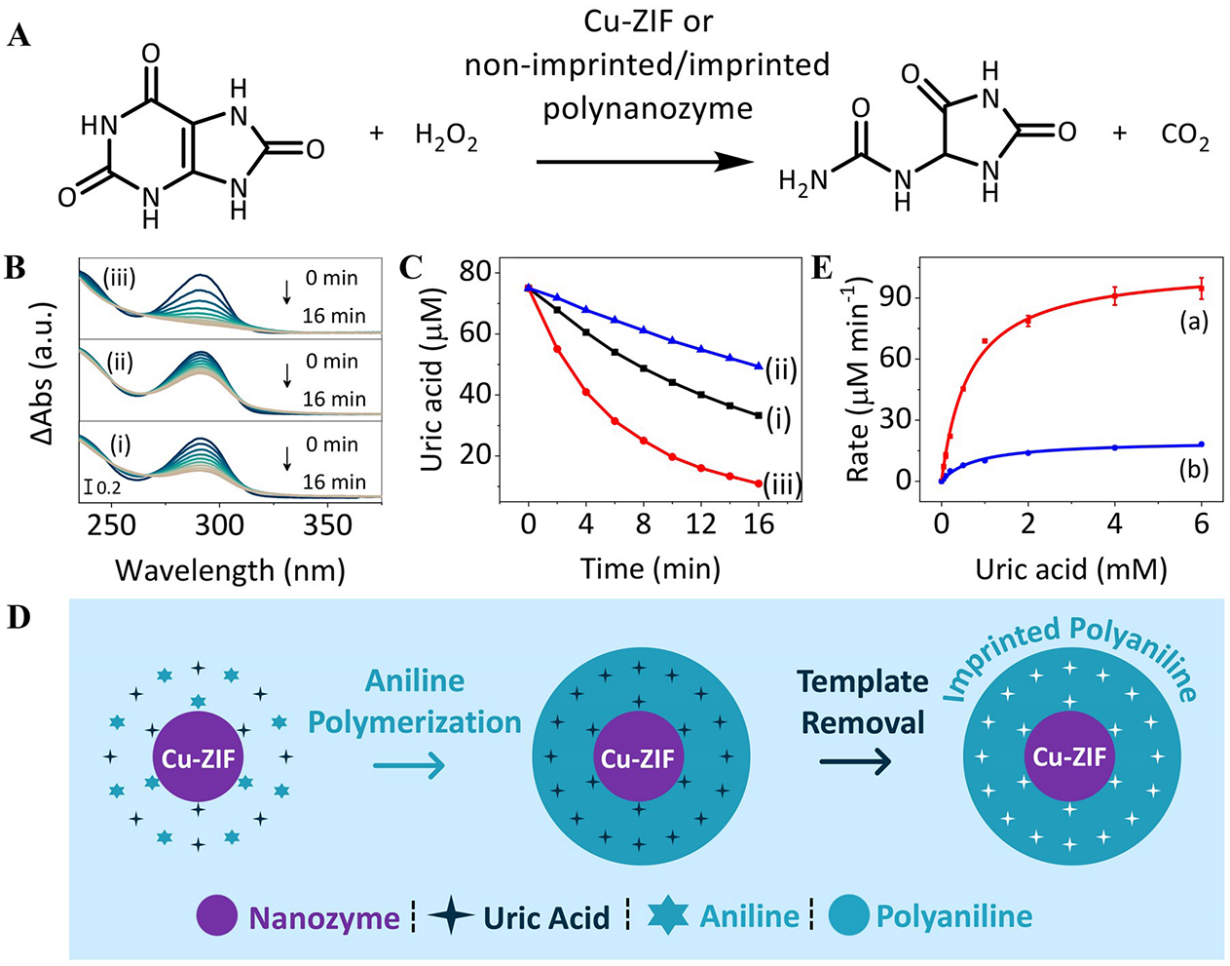
Uric acid peroxidase catalytic functions of UA-imprinted/nonimprinted PAn-coated Cu-ZIF NMOFs vs bare Cu-ZIF NMOFs. (A) Equation of the catalyzed H_2_O_2_ oxidation of UA to allantoin. (B) Absorbance spectra changes upon catalyzed H_2_O_2_ oxidation of UA by bare Cu-ZIF NMOFs (i), PAn-coated Cu-ZIF NMOFs (ii) and UA-imprinted PAn-coated Cu-ZIF NMOFs (iii). In all experiments, catalysts, 50 μg mL^–1^, UA, 75 μM, and H_2_O_2_, 5 mM, were used. (C) Time-dependent concentration changes of UA upon oxidation of UA, 75 μM, by H_2_O_2_, 5 mM, to yield allantoin in the presence of bare Cu-ZIF NMOFs, 50 μg mL^–1^ (i), PAn-coated Cu-ZIF NMOFs, 50 μg mL^–1^ (ii), and UA-imprinted PAn-coated Cu-ZIF NMOFs, 50 μg mL^–1^ (iii). (D) Schematic imprinting UA binding sites into polyaniline-coated Cu-ZIF NMOFs. (E) Rates of UA oxidation by H_2_O_2_, 5 mM, at variable concentrations of UA: (a), UA-imprinted PAn-coated Cu-ZIF NMOFs, 50 μg mL^–1^; (b), nonimprinted PAn-coated Cu-ZIF NMOFs, 50 μg mL^–1^.

We examined, first, the possible catalytic H_2_O_2_ oxidation of UA by the PAn-coated Cu-ZIF NMOFs (Figure 2A). Figure 2B, Panels (i) and (ii), shows the absorbance changes upon H_2_O_2_ oxidation of UA to allantoin by the bare Cu-ZIF NMOFs and the PAn-coated Cu-ZIF NMOFs, respectively. Figure 2C depicts the temporal concentration changes of UA upon oxidation by H_2_O_2_ yielding allantoin, in the presence of the bare Cu-ZIF NMOFs, curve (i), and the PAn-coated Cu-ZIF NMOFs, curve (ii). Evidently, the oxidation of UA to allantoin by the PAn-coated Cu-ZIF NMOFs is substantially retarded as compared to the bare Cu-ZIF NMOFs. These results are consistent with the fact that coating of the Cu-ZIF NMOFs with PAn blocks the catalytic sites of the nanozyme. Nonetheless, we argued that within the catalytic coating process of the Cu-ZIF NMOFs, we could imprint specific UA sites that serve as recognition binding sites for the association of UA in spatial proximity to the catalytic sites of the nanozyme. That is, the molecular imprinting process could yield a hybrid composite mimicking the active site/binding site cooperative functions of native enzymes, thereby providing an effective catalytic interface for the oxidation of UA. We argued that within the catalytic coating of aniline to polyaniline and in the presence of UA as a guest substrate, supramolecular interactions between the amino functionalities of PAn and amine/keto functionalities of UA could yield, after washing off of the UA, the UA molecular-imprinted sites in the PAn-coating on the Cu-ZIF NMOFs. These imprinted sites could yield a superior hybrid nanozyme catalyzing the oxidation of UA—a “polynanozyme” (Figure 2D).

Accordingly, UA was imprinted into the PAn coating of the Cu-ZIF NMOFs upon the catalyzed polymerization of aniline. Figure 2B, Panel (iii), shows the absorbance changes upon the catalyzed oxidation of UA by H_2_O_2_ to allantoin, and Figure 2C, curve (iii), depicts the temporal concentration changes upon H_2_O_2_ oxidation of UA to allantoin by the imprinted polynanozyme. In the experiment displayed in Figure 2C, curve (i–iii), the same concentrations of bare Cu-ZIF NMOFs, curve (i), PAn-coated NMOFs, curve (ii), and UA-imprinted PAn-coated NMOFs, curve (iii), were used. Figure 2E compares the rates of UA oxidation by the UA-imprinted PAn-coated Cu-ZIF NMOFs at different concentrations of UA, curve (a), with the rates of UA oxidation by the nonimprinted PAn-coated Cu-ZIF NMOFs, curve (b). As the concentration of UA increases, the rate of oxidation of UA by the imprinted PAn-coated Cu-ZIF NMOFs is higher and reaches a saturation value at a concentration of 4 mM, *V*_max_ = 106 mM min^–1^. The saturated rate is attributed to the saturation of the imprinted sites associated with the polymer coating (for estimation of the average number of imprinted sites associated with a particle, vide infra). The imprinted PAn-coated Cu-ZIF NMOFs revealed a 6.3-fold enhanced oxidation rate of UA as compared to the nonimprinted PAn-coated Cu-ZIF NMOFs. Figure S7 compares the rates of catalyzed UA oxidation by H_2_O_2_ using the bare Cu-ZIF NMOFs, the nonimprinted PAn-coated Cu-ZIF NMOFs, and the imprinted PAn-coated Cu-ZIF NMOFs at different concentrations of UA. The catalyzed H_2_O_2_ oxidation of UA by the UA-imprinted PAn-coated NMOFs reveals a ca. 4.1-fold enhancement as compared to the bare Cu-ZIF NMOFs. That is, the imprinting of molecular recognition sites into the polymer coating of the hybrid PAn/Cu-ZIF NMOFs yielded a hybrid nanozyme (“polynanozyme”), exhibiting superior catalytic functions as compared to the bare nanozyme. The superior catalytic activity of the UA-imprinted PAn-coated Cu-ZIF composite is attributed to the concentration of the UA substrate in the imprinted sites in spatial proximity to the catalytic sites of the NMOF frameworks.

**Figure 3:**
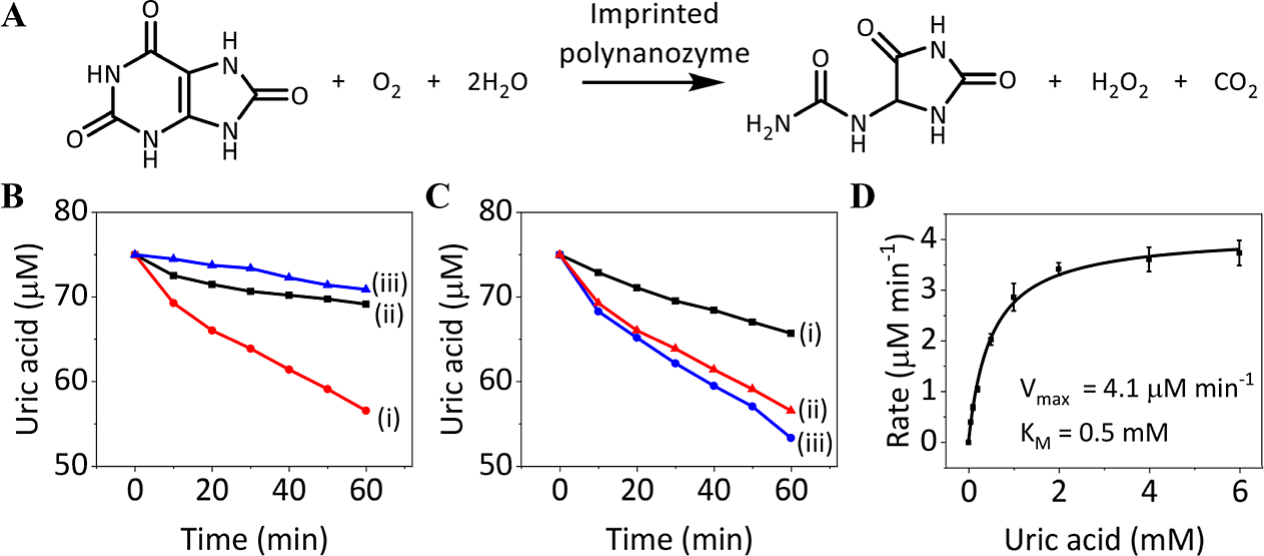
Uric acid oxidase catalytic functions of UA-imprinted/nonimprinted PAn-coated Cu-ZIF NMOFs vs bare Cu-ZIF NMOFs. (A) Equation of the catalyzed aerobic oxidation of UA to allantoin. (B) Time-dependent concentration changes of UA upon aerobic oxidation of UA, 75 μM, to allantoin in the presence of UA-imprinted PAn-coated Cu-ZIF NMOFs, 50 μg mL^–1^ (i), bare Cu-ZIF NMOFs, 50 μg mL^–1^ (ii), and nonimprinted PAn-coated Cu-ZIF NMOFs, 50 μg mL^–1^ (iii). (C) Time-dependent concentration changes of UA upon aerobic oxidation of UA, 75 μM, to allantoin in the presence of different concentrations of UA-imprinted PAn-coated Cu-ZIF NMOFs: (i) 25 μg mL^–1^; (ii) 50 μg mL^–1^; (iii) 75 μg mL^–1^. (D) Rates of aerobic oxidation of UA catalyzed by UA-imprinted PAn-coated Cu-ZIF NMOFs, 50 μg mL^–1^, in the presence of variable concentrations of UA.

In nature, the oxidation of UA to allantoin is driven by uricase under aerobic conditions, where uricase reveals oxidase (rather than peroxidase) functions (Figure 3A). In fact, native uricases are Cu^2+^-dependent enzymes or Cu^2+^-independent catalysts.^81^ Accordingly, we probed the possible functions of the Cu-ZIF NMOFs or the different PAn-coated Cu-ZIF NMOFs as possible catalysts mimicking the catalytic function of native uricase catalyzing the aerobic oxidation of UA. We find that the bare Cu-ZIF NMOFs or the PAn-coated Cu-ZIF NMOFs lack catalytic activities toward the aerobic oxidation of UA to allantoin. However, the UA-imprinted PAn-coated Cu-ZIF NMOFs reveal effective catalytic functions toward the aerobic oxidation of UA. Figure 3B shows the temporal concentration changes of UA upon aerobic oxidation of UA to allantoin in the presence of the UA-imprinted PAn-coated Cu-ZIF NMOFs, curve (i), in comparison with the reference systems, the catalyzed aerobic oxidation of UA by the bare Cu-ZIF NMOFs, curve (ii), or the nonimprinted PAn-coated Cu-ZIF NMOFs, curve (iii). While the bare NMOFs or the PAn-coated NMOFs do not show any significant catalytic function toward the aerobic oxidation of UA to allantoin, the imprinted polynanozyme reveals an oxidase catalytic activity. Figure 3C shows the temporal depletion of UA as a result of aerobic oxidation to allantoin, in the presence of a variable concentration of the imprinted polynanozyme. As the concentration of the UA-imprinted PAn-coated Cu-ZIF NMOFs increases, the aerobic oxidation of UA is enhanced. Figure 3D depicts the rates of aerobic oxidation of UA to allantoin by the UA-imprinted PAn-coated Cu-ZIF NMOFs at different concentrations of UA. The kinetic features, *K*_M_ = 0.5 mM, *V*_max_ = 4.1 μM min^–1^, of the polynanozyme were derived. The results demonstrate the assembly of a novel nanozyme hybrid structure. While the Cu-ZIF NMOFs (or PAn-coated Cu-ZIF NMOFs) lack catalytic activity toward the aerobic oxidation of UA, the hybrid nanostructure consisting of the UA-imprinted PAn-coated Cu-ZIF NMOFs reveals effective activity toward the aerobic oxidation of UA. Besides mimicking the oxidase functions of the native uricase, the imprinted polynanozyme introduces an unprecedented phenomenon in nanozyme catalysis where a hybrid structure composed of an imprinted polymer-coated nanozyme demonstrates catalytic functions that are lacking in the bare nanozyme framework.

A further important aspect related to the peroxidase/oxidase catalytic activities of the imprinted PAn-coated Cu-ZIF NMOFs involves the stability and recyclability of the polynanozyme. We find that the catalyst is stable for at least three months upon storage at 4 °C (<5% loss in activity). Moreover, Figure S10 demonstrates the repeated oxidase activity of the imprinted PAn-coated Cu-ZIF nanoparticles upon application of the same batch of particles for four repeated cycles. A gradual stepwise “loss” of the activity corresponding to ca. 20% is observed. This apparent decrease in activity is due to the loss of catalyst particles upon regeneration for the cyclic process. In fact, keeping the polynanozyme nanoparticles in an aqueous solution for at least one month at room temperature does not affect their activity.

### Selectivity of Peroxidase/Oxidase Activities of the UA-Imprinted PAn-Coated Cu-ZIF NMOFs

The imprinting of UA binding sites in the PAn polymer coating suggests that selectivity toward binding of the UA substrate should exist. Moreover, the quantitative assessment of the average number of imprinted sites should be considered. Figure S11 and the accompanying discussion present the method to estimate the average number of imprinted sites associated with the imprinted PAn coating of a single NMOF particle. We find that about 6.45 × 10^9^ imprinted sites per particle are generated. The shape selectivity issue associated with the UA imprinted sites and the oxidation of UA is, however, an important, yet difficult issue to address since we could not identify a UA structural analogue that can be oxidized by the Cu-ZIF NMOFs. Nevertheless, hypoxanthine could provide a shape analogue of UA that could bind to the imprinted sites. Although the hypoxanthine is not oxidized by the imprinted PAn-coated Cu-ZIF NMOFs in the presence of H_2_O_2_ or O_2_, we find that hypoxanthine significantly inhibits the catalyzed oxidation of UA by polynanozyme, Figure S12A,B. That is, competitive binding of hypoxanthine to the UA-imprinted sites inhibits binding of the UA substrate to the catalyst coating, thus deconcentrating the UA shape-like binding sites present within the polymer coating. Moreover, the distribution of the imprinted sites on the polymer coating has a significant effect on the activity of the resulting polynanozyme. As the concentration of the imprinted sites is controlled by the concentration of UA employed in the imprinting process, the catalytic performance of the polynanozyme should be dictated by the distribution and content of the imprinted sites within the polymer coating. This is demonstrated in Figure S13, where the imprinting process was performed in the presence of variable concentration of the imprinting ligand.

### Mechanistic Pathways Associated with the Cu-ZIF NMOFs and Polynanozymes

**Figure 4:**
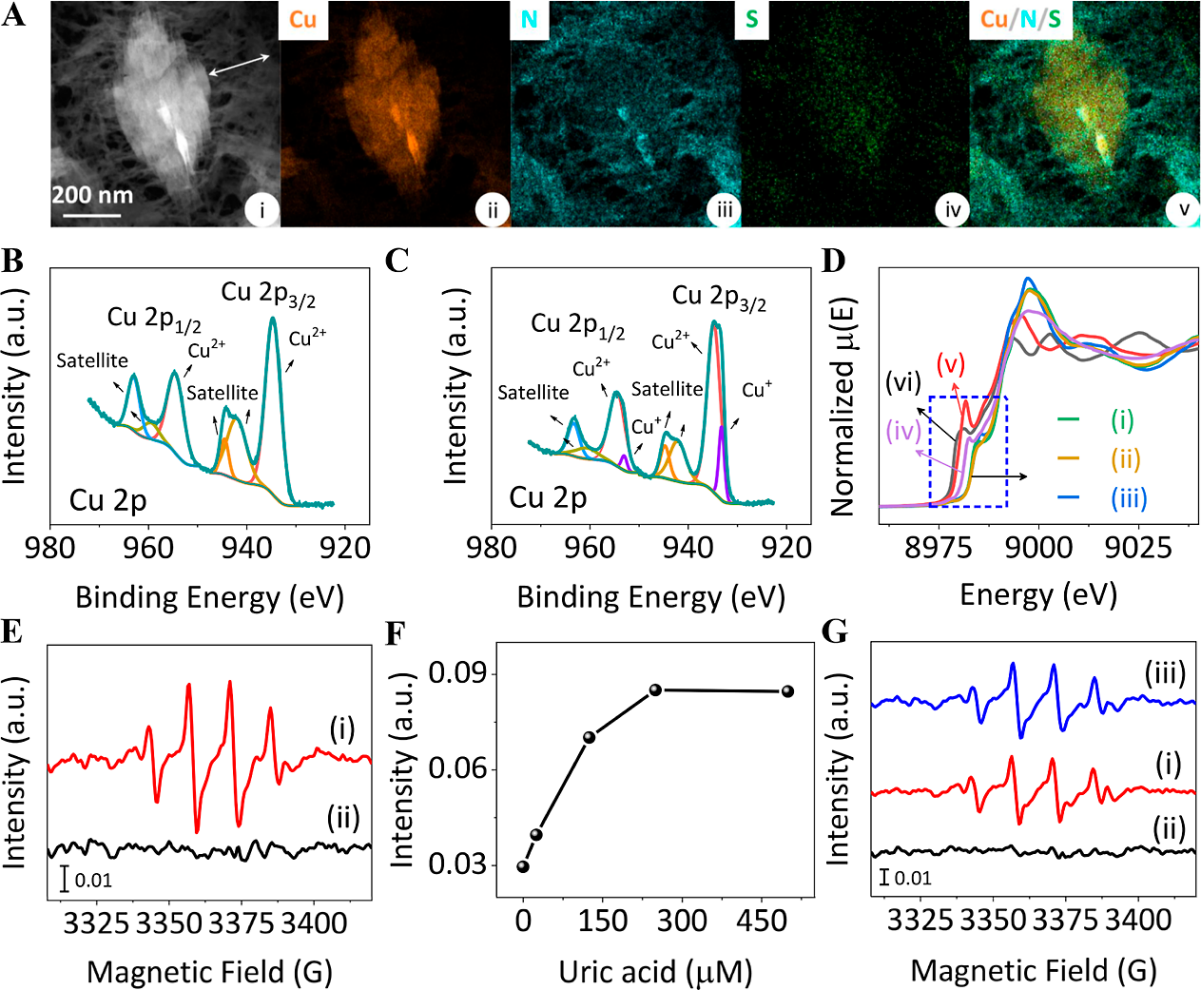
Compositional and functional features of UA-imprinted PAn-coated Cu-ZIF NMOFs in comparison with bare Cu-ZIF NMOFs. (A) HAADF-STEM image (i) and accompanying EDS element mapping images of the UA-imprinted PAn-coated Cu-ZIF NMOFs: (ii) Cu; (iii) N; (iv) S; (v) overlay of (ii–iv). (B,C) Deconvoluted Cu 2p XPS spectra of the nonimprinted PAn-coated Cu-ZIF NMOFs (B) and the UA-imprinted PAn-coated Cu-ZIF NMOFs (C). (D) Cu K-edge XANES spectra of the bare Cu-ZIF NMOFs (i), nonimprinted PAn-coated Cu-ZIF NMOFs (ii), CuO (iii), UA-imprinted PAn-coated Cu-ZIF NMOFs (iv), Cu_2_O (v), and Cu foil (vi). (E) EPR spectra generated by the UA-imprinted PAn-coated Cu-ZIF NMOFs in the presence of air and added BMPO, 25 mM (i), and by the UA-imprinted PAn-coated Cu-ZIF NMOFs in the presence of air, BMPO, 25 mM, and added superoxide dismutase, SOD (ii). (F) Intensities of the EPR spectra at 3357 G generated by the UA-imprinted PAn-coated Cu-ZIF NMOFs in the presence of added BMPO, 25 mM, and variable concentrations of UA under air. (G) EPR spectra generated by the bare Cu-ZIF NMOFs under aerobic conditions and added BMPO, 25 mM, in the absence of UA (i), in the absence of UA and added SOD (ii), and in the presence of UA, 250 μM, and in the absence of SOD (iii).

To further characterize the system, a comprehensive set of microscopic, spectroscopic, and mechanistic studies were performed. The high-angle annular dark field scanning transmission (HAADF-STEM) image and the accompanying energy-dispersive X-ray spectroscopy (EDS) element mapping images of UA-imprinted PAn-coated Cu-ZIF NMOFs are displayed in Figure 4A (for analogue images of the bare Cu-ZIF NMOFs and the nonimprinted PAn-coated Cu-ZIF NMOFs, see Figures S14 and S15, respectively). The UA-imprinted PAn-coated Cu-ZIF NMOFs, Panel (i), reveal a length of ca. 740 nm and a width of ca. 330 nm, which is slightly larger than the length/width of the bare Cu-ZIF NMOF flake, Figure S14, Panel (i), consistent with the coating of the particles by PAn. The BET specific surface area of the UA-imprinted PAn-coated Cu-ZIF NMOFs corresponds to 797.0 m^2^ g^–1^ (Figure S16). The coating of the NMOFs by imprinted PAn is further supported by the morphological mapping of the elements composing the hybrid particles. The core of the particle, Panel (ii), shows the Cu constituent. Panels (iii and iv) show the distribution of the N and S elements throughout the particle coating, consistent with the formation of the poly(styrenesulfonate)-integrated PAn coating. Panel v depicts the merged image of the constitutional elements composing the hybrid NMOFs. A clear core Cu-element domain, coated with the polymer coating, about 212 nm thick, is visible, supporting the constituents composing the hybrid structure (for comparison, the element mapping images of the bare Cu-ZIF NMOFs, Figure S14, reveal only the core Cu/N elements with no contribution of the elements composing the coating domain). The XPS results corresponding to the Cu 2p of the Cu-ZIF NMOFs, the PAn-coated Cu-ZIF NMOFs, and the UA-imprinted PAn-coated Cu-ZIF NMOFs are displayed in Figures 1B and 4B,C, respectively (for the XPS spectra of the other elements composing the PAn-coated Cu-ZIF NMOFs and the UA-imprinted PAn-coated Cu-ZIF NMOFs, see Figures S17 and S18). While the bare Cu-ZIF NMOFs and the PAn-coated Cu-ZIF NMOFs demonstrate the existence of Cu^2+^ in the structure, the UA-imprinted PAn-coated NMOFs show a Cu^2+^/Cu^+^ composition in the NMOF structure. That is, presumably, during the UA imprinting process of the PAn-coating on the NMOFs, the Cu^2+^ composing the NMOFs is partially reduced to yield a Cu^2+^/Cu^+^ mixture (87.30%/12.70%) in the NMOF framework. The copper valences in the bare Cu-ZIF NMOFs, the PAn-coated Cu-ZIF NMOFs, and the UA-imprinted PAn-coated Cu-ZIF NMOFs were further evaluated by Cu K-edge X-ray absorption fine structure (XAFS) measurement. The X-ray absorption near edge structure (XANES) spectra in Figure 4D display that the adsorption edge of the Cu-ZIF nanozyme, curve i, and the PAn-coated Cu-ZIF NMOFs, curve ii, overlay the adsorption edge of the reference CuO sample, curve (iii), indicating that copper in the bare NMOFs and the PAn-coated NMOFs exhibits pure Cu^2+^ valence. In turn, the absorption edge of the UA-imprinted NMOFs, curve (iv), is shifted and positioned between the reference CuO, curve (iii), and Cu_2_O, curve (v), implying that the copper in the UA-imprinted PAn-coated NMOFs includes a mixture of Cu^2+^ and Cu^+^ valences, which is consistent with the XPS measurements. Indeed, the existence of Cu^+^ in the framework assists us in understanding the catalytic activity of the UA-imprinted PAn-coated Cu-ZIF NMOFs toward the aerobic oxidation of UA by the polynanozyme (vide infra). In addition, the significance of the Cu^+^ constituent in the aerobic oxidation of UA is further demonstrated by a control experiment in which the Cu^+^, generated within the imprinted process is chemically depleted, Figure S19. In the experiment, the as-prepared imprinted PAn-coated hybrid NMOFs (at the Cu^2+^/Cu^+^ ratio: 87.3%/12.7%) were oxidized with Na_2_S_2_O_8_ yielding a hybrid with negligible Cu^+^ content. As is evident from Figure S20, the oxidase activity of the resulting NMOFs was significantly inhibited, demonstrating the significance of the Cu^+^ units in the aerobic oxidation of UA.

Moreover, the kinetic results demonstrating the superior peroxidase activity of the UA-imprinted PAn-coated Cu-ZIF NMOFs toward the oxidation of UA by H_2_O_2_, as compared to the substantially lower activities of the bare Cu-ZIF NMOFs or the nonimprinted PAn-coated NMOFs, were attributed to the binding affinity of UA to the imprinted sites and the spatial concentration of UA in proximity to the Cu^2+^-catalytic sites (“molarity effect”). Indeed, isothermal titration calorimetry (ITC) measurements confirmed the binding affinity of UA for the imprinted PAn-coated NMOFs, Figure S21. While the bare Cu-ZIF NMOFs or the PAn-coated NMOFs did not show binding affinities toward the UA, Figure S21A–D, the imprinted PAn-coated NMOFs revealed a high binding affinity curve, *K*_D_ ∼ 120 μM, Figure S21E,F, consistent with the presence of imprinted binding sites for UA in the hybrid framework.

**Figure 5:**
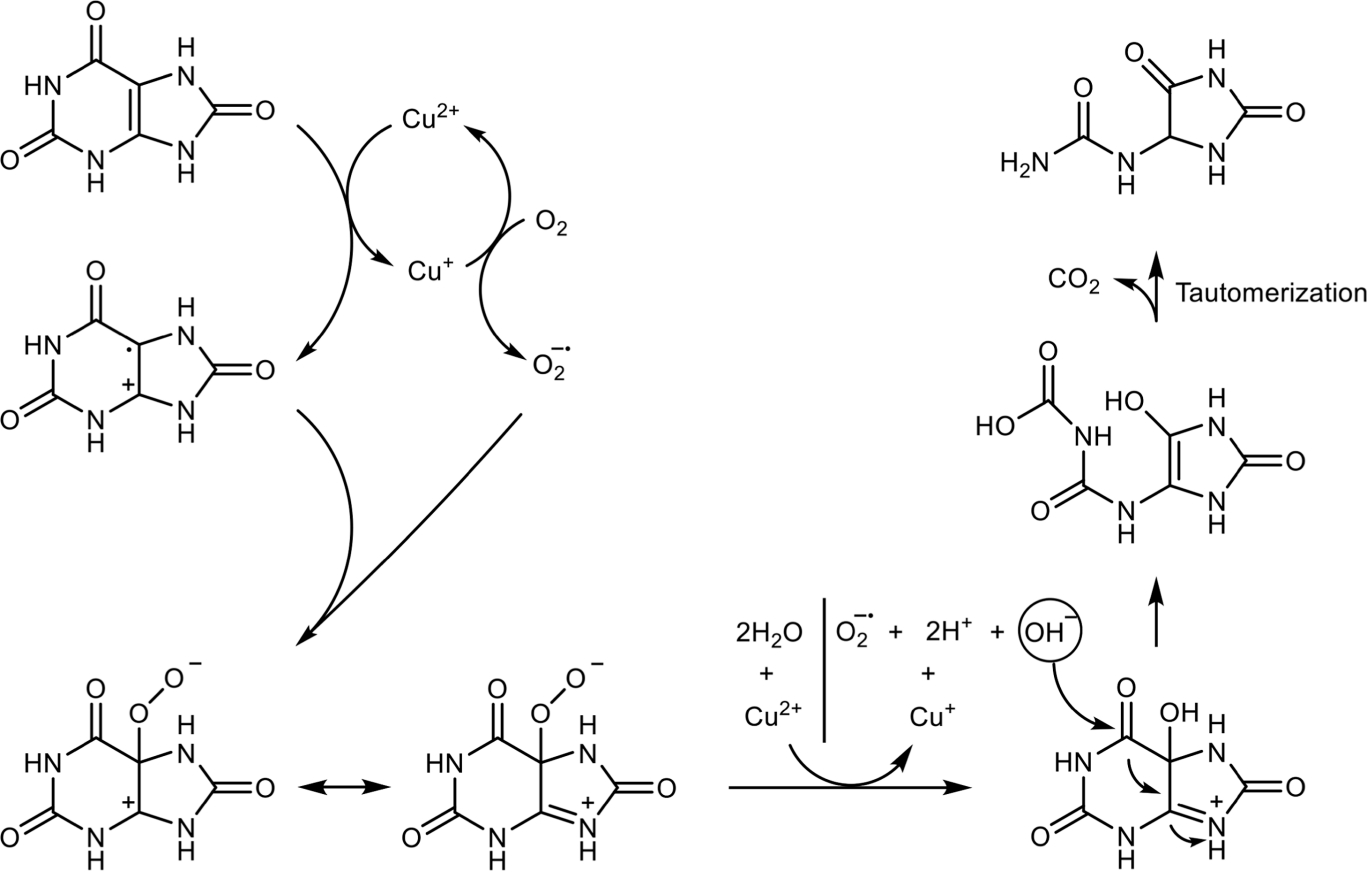
Suggested mechanism for the aerobic oxidation of UA to allantoin by the UA-imprinted PAn-coated Cu-ZIF NMOFs.

Finally, mechanistic aspects related to the oxidation of UA in the presence of H_2_O_2_ or O_2_ are introduced. Particularly, the discovery that the UA-imprinted PAn-coated Cu-ZIF NMOFs are effective catalysts catalyzing the aerobic oxidation of UA to allantoin, while the bare catalyst lacks this catalytic activity, is addressed. In fact, previous reports suggested possible mechanistic paths for the aerobic oxidation of UA by uricase, and participation of the reactive superoxide radical (O_2_^–•^) intermediate was suggested,^82,83^ and recently, the participation of O_2_^–•^ in the aerobic oxidation of UA by nanozyme was reported.^84,85^ In the present study, we introduce experimental data that provide insight into the mechanism of UA oxidation under aerobic conditions or in the presence of H_2_O_2_, and we suggest a possible mechanistic path for the oxidation processes. First, we address the aerobic oxidation of UA by the UA-imprinted PAn-coated Cu-ZIF NMOFs. Figure 4E shows that the UA-imprinted PAn-coated NMOFs yield in the presence of air a ROS product that can be assigned to O_2_^–•^ [curve (i)]. The formation of O_2_^–•^ is confirmed by the addition of superoxide dismutase (SOD) that depletes the signal, curve (ii). Furthermore, the electron paramagnetic resonance (EPR) signal of O_2_^–•^ increases in the presence of added UA, Figures 4F and S22, suggesting that it participates in the catalytic formation of O_2_^–•^ (vide infra). The nonimprinted PAn-coated NMOFs lead to a negligible O_2_^–•^ signal, implying that the catalytic activity toward the generation of O_2_^–•^ is inhibited. In turn, the bare Cu-ZIF NMOFs show a substantially lower EPR intensity of the O_2_^–•^, suggesting lower catalytic activity toward the generation of the O_2_^–•^, and no effect of UA on the signal was observed, Figure 4G. The effective formation of O_2_^–•^ by the imprinted PAn-coated NMOFs suggests that oxygen was slowly reduced by a constituent in the system. Moreover, toward the significance of O_2_^–•^ as a reactive intermediate in the catalyzed aerobic oxidation of UA by the imprinted PAn-coated Cu-ZIF NMOFs, we examined the reaction in the presence of different concentrations of SOD that depletes the O_2_^–•^, Figure S23. Evidently, as the concentration of SOD increases, enhanced inhibition of the process is observed, and at an SOD concentration of 0.2 units L^–1^, the process is fully inhibited. To summarize the results presented in this section, we emphasize the following issues: (i) The XPS and XANES experiments (Figures 1B and 4B–D) demonstrated the presence of Cu^+^ as a coconstituent in the imprinted polynanozyme, yet this constituent is absent in the nonimprinted PAn-coated particles or the bare particles. (ii) The EPR experiments indicated that under aerobic conditions, O_2_^–•^ were formed in the imprinted polynanozyme-catalyzed reaction. Moreover, the results indicated that the content of the O_2_^–•^ depended on the concentration of added UA, and at higher UA concentrations, the content of the O_2_^–•^ increases, Figure 4E,F, suggesting that UA coparticipated in the formation of the O_2_^–•^. (iii) The links between the O_2_^–•^ and the catalyzed aerobic oxidation of UA by the imprinted polynanozyme were demonstrated by the inhibition of the process with SOD, Figure S23. (iv) The intermediate formation of the UA^+^ within the aerobic oxidation of UA by the imprinted polynanozyme was suggested by the EPR experiment, Figure S24. This analysis allows us to formulate a possible pathway for the oxidation of UA to allantoin by the imprinted PAn-coated Cu-ZIF NMOFs, Figure 5. The suggested mechanism includes the concomitant generation of O_2_^–•^ by the Cu^+^ constituent generated within the imprinting procedure and by the UA^+^ generated by the catalyzed UA oxidation by the imprinted PAn-coated Cu-ZIF NMOFs. The addition of O_2_^–•^ to the UA^+^ yields a peroxy radical intermediate where the peroxy constituent reduces Cu^2+^ to Cu^+^, and concomitantly, the intermediate radical is oxidized by Cu^2+^ to form the hydroxy-substituted UA that is hydrolytically ring-opened to carboxycarbonate undergoing decarboxylation and tautomerization, yielding the allantoin product.

The participation of UA in the formation of O_2_^–•^ and the efficient aerobic oxidation of UA catalyzed by the imprinted PAn-coated Cu-ZIF NMOFs polynanozyme still raise some questions: (i) Why is the activity of the bare Cu-ZIF NMOFs negligibly low? (ii) What causes the polynanozyme hybrid to be highly active in catalyzing the oxidation of UA? We reason that within the imprinting procedure of the UA sites, a high concentration of UA exists in close proximity to the Cu-ZIF framework, leading to the formation of O_2_^–•^. The formation of O_2_^–•^ in the first step of the mechanism shown in Figure 5 is due to the primary Cu^2+^ oxidation of UA to UA^+^ and the concomitant formation of Cu^+^ that reduces O_2_ to O_2_^–•^. The concentration of UA at the particle sites enhances this electron transfer process, yielding the crucial Cu^+^ intermediate, which is the key intermediate for the oxidase activity of the polynanozyme. (Note that the appearance of Cu^+^ in the framework was confirmed by XPS and XANES only in the imprinted NMOFs and further suggested by the relation of the Cu^+^ content and the concentration of UA in the imprinted sites, see Figure S25.) The resulting O_2_^–•^ is presumably stabilized by hydrogen bonds within the PAn framework, thereby being protected from diffusing to the bulk solution by the hybrid structure. Accordingly, in the presence of the imprinted PAn hybrid NMOFs framework, a functional nanoparticle module for the concentration of the UA in the imprinted sites is formed, allowing the effective process outlined in Figure 5 to proceed where efficient UA-driven formation and stabilization of the oxidation of the O_2_^–•^ in the supramolecular nanostructure assembly is followed by the oxidation of UA, yielding allantoin. These conditions are absent in the diffuse configuration of the bare NMOFs, where the diluted O_2_^–•^ and UA result in a very low level of oxidation of UA and competitive depletion of the unstable O_2_^–•^ reactant.

Moreover, the peroxidase activities of the catalyzed oxidation of UA by H_2_O_2_ are further rationalized by the reaction outlined in eq 2. Realizing that the O_2_^–•^ is generated by the reaction between Cu-ZIF NMOFs and H_2_O_2_, Figure S26, the catalyzed oxidation of H_2_O_2_ by the Cu-ZIF NMOFs to form O_2_^–•^ and the formation of Cu^+^, eq 2, is suggested as a key step in the peroxidase activity of the nanozyme.^86^ The resulting Cu^+^ can further react with oxygen as described before or be depleted by H_2_O_2_. However, the O_2_^–•^ generated following eq 2 is suggested as the main source acting as the active reaction intermediate participating in the oxidation of UA, following multistep reactions suggested in Figure 5. The effective peroxidase activity of the imprinted polynanozyme is, then, attributed to the stabilization of O_2_^–•^ at the catalytic interface by the polymer coating, and the concentration of the UA substrate within the imprinted sites.
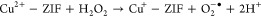
2

## Conclusions

The peroxidase functions of the Cu-ZIF metal–organic framework nanoparticles, Cu-ZIF NMOFs, toward the catalyzed H_2_O_2_ oxidation of uric acid, UA, to allantoin were introduced. In addition, the Cu-ZIF NMOFs catalyzed the H_2_O_2_ oxidation of aniline to polyaniline, PAn, and the coating of the NMOFs with the PAn was demonstrated. The latter process was employed to imprint UA molecular binding sites into the polymer coating. While the nonimprinted PAn-coated Cu-ZIF NMOFs were inhibited toward the catalyzed oxidation of UA by H_2_O_2_, the UA-imprinted PAn-coated Cu-ZIF NMOFs hybrid revealed enhanced catalytic activities toward the catalyzed H_2_O_2_ oxidation of UA. The enhanced catalytic activities of the imprinted PAn-coated Cu-ZIF NMOFs were attributed to the binding and concentration of the UA substrate by the imprinted sites in close proximity to the catalytic sites of Cu-ZIF NMOFs (“molarity effect”). The binding affinity of UA for the imprinted sites was confirmed by ITC measurements. Moreover, the imprinted PAn-coated Cu-ZIF NMOFs, polynanozymes, were demonstrated with the function of catalyzing the aerobic oxidation of UA to allantoin. While the imprinted PAn-coated Cu-ZIF NMOFs demonstrated effective catalytic aerobic oxidation of UA, the bare Cu-ZIF NMOFs showed negligible catalytic functions toward the aerobic oxidation of UA. This is an unprecedented example where the imprinted NMOFs hybrid demonstrated catalytic functions absent in the bare nanozyme. Moreover, attempts to characterize the mechanistic pathway involved with the oxidase and peroxidase activities of the imprinted polynanozyme revealed the participation of superoxide radicals, O_2_^–•^, as active reaction intermediates, allowing us to suggest possible mechanistic circuits for the catalytic oxidase/peroxidase catalytic processes. While the results introduce new dimensions to the area of nanozymes, important challenges are ahead of us. The development of other imprinted PAn-coated nanozymes, such as other NMOFs or metal-functionalized carbon nanomaterials, and particularly the development of other nanozyme-catalyzed imprinted polymer coatings, such as polypyrrole or polypyridine, are certainly important paths to follow. Moreover, the application of the imprinted polynanozymes to catalyze other catalytic transformations and particularly to identify practical uses of the composites would be interesting scientific goals.

## Experimental Methods

### Chemicals

Copper(II) chloride dihydrate (CuCl_2_·2H_2_O), hexadecyltrimethylammonium bromide, 2-methylimidazole, uric acid (UA, ε_292 nm_ = 12,600 M^–1^ cm^–1^), hydrogen peroxide solution (H_2_O_2_, 30% w/w), aniline (purified by steam-distillation before use), superoxide dismutase from bovine (SOD, ≥ 2500 units/mg protein), and phosphate-buffered saline (PBS) were purchased from Sigma-Aldrich (USA). Poly(styrene sulfonic acid sodium salt) (MW 70,000) was obtained from Polysciences, Inc. (USA). 5-Tert-butoxycarbonyl-5-methyl-1-pyrroline-*N*-oxide (BMPO) was bought from Dojindo laboratories (Japan). *P*-(3,4-Dihydro-2-methyl-1-oxido-2*H*-pyrrol-2-yl)-phosphonic acid, diethyl ester (DEPMPO) was purchased from Cayman Chemical (USA). All the chemicals were used without further purification unless otherwise specified. Ultrapure water (18.2 MΩ cm) from NANOpure Diamond (Barnstead Int., USA) was used in all the experiments.

### Characterizations

The UV–vis absorption spectra were measured by a UV-1900 UV–vis spectrophotometer (Shimadzu, Japan), and quartz cuvettes with path lengths of 1 and 0.3 cm were used for the spectra and Michaelis–Menten kinetic features, respectively. The scanning electron microscopy (SEM) images were taken from a cryo high-resolution scanning electron microscope Apreo 2S (Thermo Fisher Scientific, USA). The transmission electron microscopy (TEM) image, high-resolution TEM (HR-TEM) images, high-angle annular dark field scanning transmission (HAADF-STEM) images, and the accompanying energy-dispersive X-ray spectroscopy (EDS) element mapping images were taken on an aberration probe-corrected scanning transmission electron microscope Themis Z (Thermo Fisher Scientific, USA). The powder X-ray diffraction (PXRD) spectrum was obtained on an X-ray powder diffractometer D8 ADVANCE with a 2.2 kW Cu Kα X-ray source mounted into the TWIST-TUBE assembly (Bruker, USA). The N_2_ adsorption–desorption (77 K) isotherms were measured by a NOVA1200e (Quantachrome, USA). The X-ray photoelectron spectroscopy (XPS) spectra were collected by a Kratos AXIS Supra spectrometer with an Al Kα monochromatic radiation X-ray source (1486.6 eV) as the excitation source (Kratos, UK). The X-ray absorption near-edge structure (XANES) spectra were collected at room temperature in the transmission mode at the Shanghai synchrotron radiation facility (SSRF, China). The dissociation constants of the UA to the catalysts were calculated based on the measured heat difference during the binding events by isothermal titration calorimetry (ITC) on a MicroCal PEAQ-ITC (Malvern Panalytical, UK), and one set of site fitting models was used. The particle number was counted with a TC20 automated cell counter (USA). The electron paramagnetic resonance (EPR) spectra were taken on a Bruker ELEXSYS 500 X-band spectrometer equipped with a Bruker ER4102ST resonator at room temperature with a microwave power of 20 mW, a 0.1 G modulation amplitude, and a 100 kHz modulation frequency (Bruker, USA).

### Synthesis of Cu-ZIF NMOFs

The Cu-ZIF NMOFs were prepared in the aqueous condition following the reported procedure^58^ with slight modifications. Briefly, 2-methylimidazole (1 M, 10 mL) and hexadecyltrimethylammonium bromide (1 mM, 10 mL) were added simultaneously to copper(II) chloride (50 mM, 10 mL) under stirring conditions (300 rpm) at RT. After being stirred for 1 min, the solution was left unstirred for 45 min. Then, the brownish precipitates were collected and purified by centrifugation (7000 rcf, 15 min). The resulting products were redispersed with ultrapure water (8 mL) and stored at 4 °C. To evaluate the concentration of the products, 1 mL of the dispersions was freeze-dried, and the residue was weighed.

### Synthesis of PAn-Coated Cu-ZIF NMOFs and UA-Imprinted PAn-Coated Cu-ZIF NMOFs

For the synthesis of PAn-coated Cu-ZIF NMOFs or UA-imprinted PAn-coated Cu-ZIF NMOFs, aniline (120 mM, 50 μL), poly(styrenesulfonate) (120 mM, 50 μL), PBS (200 mM, 50 μL, pH 7.4), and ultrapure water (120 μL) were was first mixed with stirring (300 rpm) for 5 min at RT. Then, Cu-ZIF NMOF dispersions (5 mg mL^–1^, 100 μL) were added with stirring (300 rpm) for 10 min, followed by adding 80 μL of ultrapure water (80 μL of UA, 50 mM, instead of ultrapure water to synthesize the UA-imprinted PAn-coated Cu-ZIF NMOFs). The catalyzed polymerization and coating (or imprinting) processes were initiated by the addition of H_2_O_2_ (200 mM, 50 μL), and the reaction was left under constant stirring for 4 h. The PAn-coated Cu-ZIF NMOFs or UA-imprinted PAn-coated Cu-ZIF NMOFs were finally obtained after purification by centrifugation (12,000 rpm, 10 min) with ultrapure water (2 mL each time) 4 times. The UA-imprinted PAn-coated Cu-ZIF NMOFs obtained with different concentrations of UA were synthesized following a similar procedure (note: the UA-imprinted PAn-coated Cu-ZIF discussed throughout the paper was synthesized with 8 mM UA unless otherwise specified). The particle number of UA-imprinted PAn-coated Cu-ZIF NMOFs was determined by using a TC20 automated cell counter. To estimate the average number of imprinted sites associated with the imprinted PAn coating of a single particle, 50 mM UA was incubated with variable concentrations of particles (0.5 mg, 1 mg, and 1.5 mg particles in 200 mL of solution) at a N_2_ condition for 5 min. Subsequently, gentle centrifugation was performed, and the absorbance of the supernatant was measured to calculate the concentration of the residual UA. The number of imprinted sites of a single particle was estimated by calculating the number of UA molecules associated with the imprinted sites of the particle.

### Catalytic H_2_O_2_ Oxidation of Aniline to Polyaniline by Cu-ZIF NMOFs

The catalytic activity and kinetic feature of Cu-ZIF NMOFs toward the H_2_O_2_ oxidation of aniline to polyaniline were monitored by the time-dependent absorbance changes of polyaniline. Briefly, PBS (pH 7.4), aniline, poly(styrenesulfonate), H_2_O_2_, and Cu-ZIF NMOFs were mixed in sequence with the final concentrations of 10 mM, 1.2 mM, 1.2 mM, 2 mM, and 100 μg mL^–1^, respectively. Repeat-scan mode with a time interval of 10 min was carried out immediately to record the absorbance spectra of obtained polyaniline by a UV-1900 UV–vis spectrophotometer at RT. For better presentation, the background absorbance of the Cu-ZIF NMOFs was subtracted from the results.

### Catalytic H_2_O_2_ Oxidation of Uric Acid to Allantoin by Cu-ZIF NMOFs, PAn-Coated Cu-ZIF NMOFs, and UA-Imprinted PAn-Coated Cu-ZIF NMOFs

Typically, PBS (pH 7.4), UA, H_2_O_2_, and Cu-ZIF NMOFs were mixed in sequence with the final concentrations of 10 mM, 75 μM, 5 mM, and 50 μg mL^–1^, respectively. Immediately, repeat-scan mode with a time interval of 2 min was started to record the time-dependent absorbance changes upon the Cu-ZIF NMOF-catalyzed oxidation of UA by H_2_O_2_ using a UV-1900 UV–vis spectrophotometer at RT. The time-dependent absorbance changes upon oxidation of UA to allantoin by H_2_O_2_ in the presence of variable concentrations of the Cu-ZIF NMOFs (25, 75 μg mL^–1^) were conducted with the same procedure. The catalytic activities of PAn-coated Cu-ZIF NMOFs and UA-imprinted PAn-coated Cu-ZIF NMOFs toward the H_2_O_2_ oxidation of UA to allantoin were monitored following the same procedure with the final concentrations of PBS (pH 7.4), UA, H_2_O_2_, and polynanozyme to be 10 mM, 75 μM, 5 mM, and 50 μg mL^–1^, respectively. For a better presentation, the background absorbance of the Cu-ZIF NMOFs was subtracted from the results.

The Michaelis–Menten kinetics feature of Cu-ZIF NMOFs, PAn-coated Cu-ZIF NMOFs, and UA-imprinted PAn-coated Cu-ZIF NMOFs toward the H_2_O_2_ oxidation of UA to allantoin were performed by measuring the absorbance changes at 292 nm in the presence of variable concentrations of UA (0 μM, 50 μM, 100 μM, and 200 μM) by kinetic mode using a UV-1900 UV–vis spectrophotometer at RT. The concentrations of PBS (pH 7.4), H_2_O_2_, and catalysts are 10 mM, 5 mM, and 50 μg mL^–1^, respectively. The initial rates were calculated by the slope of the initial linear portion of the kinetic curve. The initial rates of the catalyzed H_2_O_2_ oxidation of UA to allantoin by these nanozymes at high concentrations of UA (500 μM, 1000 μM, 2000 μM, 4000, μM, and 6000 μM) were obtained by incubating the reaction solution in a tube and then centrifuging it at fixed time intervals. Immediately, the resulting supernatant was diluted to measure the absorbance of UA and the oxidation rates were evaluated by the time-dependent concentration changes of UA. The plot of initial rates against the concentrations of UA was fitted to the Michaelis–Menten equation to get *K*_M_ and *V*_max_.

### Catalytic Aerobic Oxidation of Uric Acid to Allantoin by Cu-ZIF NMOFs, PAn-Coated Cu-ZIF NMOFs, and UA-Imprinted PAn-Coated Cu-ZIF NMOFs

PBS (pH 7.4), UA, and catalysts were mixed in sequence with final concentrations of 10 mM, 75 μM, and 50 μg mL^–1^, respectively. Immediately, repeat-scan mode with a time interval of 10 min was started to record the time-dependent absorbance changes upon the catalyzed aerobic oxidation of UA using a UV-1900 UV–vis spectrophotometer at RT. The time-dependent absorbance changes upon the aerobic oxidation of UA to allantoin in the presence of variable concentrations of the UA-imprinted PAn-coated Cu-ZIF NMOFs (25 μg mL^–1^, 75 μg mL^–1^) were conducted with the same procedure. For a better presentation, the background absorbance of the Cu-ZIF NMOFs was subtracted from the results.

The Michaelis–Menten kinetics feature of UA-imprinted PAn-coated Cu-ZIF NMOFs toward the aerobic oxidation of UA to allantoin was performed by measuring the absorbance changes at 292 nm in the presence of variable concentrations of UA (0 μM, 25 μM, 50 μM, 100 μM, 150 μM, 200 μM, 250 μM, and 300 μM) by kinetic mode using a UV-1900 UV–vis spectrophotometer at RT. The concentrations of PBS (pH 7.4) and UA-imprinted PAn-coated Cu-ZIF NMOFs are 10 mM and 50 μg mL^–1^, respectively. The initial rates were calculated by the slope of the initial linear portion of the kinetic curve. The initial rates of the catalyzed aerobic oxidation of UA to allantoin by UA-imprinted PAn-coated Cu-ZIF NMOFs at high concentrations of UA (500 μM, 1000 μM, 2000 μM, 4000 μM, and 6000 μM) were obtained by incubating the reaction solution in a tube and then centrifuging it at a fixed time interval. Immediately, the resulting supernatant was diluted to measure the absorbance of UA and the rates of UA oxidation were evaluated by the time-dependent concentration changes of UA. The plot of initial rates against the concentration of UA was fitted to the Michaelis–Menten equation to get *K*_M_ and *V*_max_.

### Binding Affinity of Uric Acid to Cu-ZIF NMOFs, PAn-Coated Cu-ZIF NMOFs, and UA-Imprinted PAn-Coated Cu-ZIF NMOFs

The binding affinity (*K*_D_) of UA to Cu-ZIF NMOFs, PAn-coated Cu-ZIF NMOFs, and UA-imprinted PAn-coated Cu-ZIF NMOFs was calculated based on the measured heat difference during binding events on a MicroCal PEAQ-ITC. 280 μL of catalyst dispersions (100 μg mL^–1^) in PBS (pH 7.4, 100 mM) and 280 μL of the same buffer were loaded in the sample cell and the reference cell, respectively. 40 μL of fresh UA (5 mM) in the same buffer was loaded in the syringe. Then, the titration curve was recorded at 25 °C by injecting UA (13 injections) into the sample cell with an injection volume of 3 μL (0.4 μL for the first injection), an injection duration of 6 s (0.8 s for the first injection), an injection spacing of 150 s, a reference power of 41.9 μW, and a stirring speed of 750 rpm. The control sample was measured by injecting UA into the sample cell containing only the same buffer following the same procedure. To calculate the *K*_D_ of UA to the catalysts, the resulting isotherm of the control sample was subtracted by the point-to-point method from the resulting isotherm of the samples, and one set of site fitting models was used.

### Selectivity of Peroxidase/Oxidase Activities of UA Imprinted PAn-Coated Cu-ZIF NMOFs

The selectivity of peroxidase/oxidase activities of UA-imprinted PAn-coated Cu-ZIF NMOFs was studied by competitive binding of hypoxanthine to the UA-imprinted sites. PBS (pH 7.4), hypoxanthine, and Cu-ZIF NMOFs were mixed in sequence with the final concentrations of 10 mM, 75 μM (or 375 μM), and 50 μg mL^–1^, respectively. After incubating for 10 min, UA and H_2_O_2_ (no addition in the case of the aerobic oxidation of UA) were added with the final concentrations of 75 μM and 5 mM, respectively. Immediately, repeat-scan mode with a time interval of 2 min (10 min, in the case of aerobic oxidation of UA) was started to record the time-dependent absorbance changes upon the Cu-ZIF NMOF-catalyzed oxidation of UA by H_2_O_2_ using a UV-1900 UV–vis spectrophotometer at RT.

### Identification of Reactive Oxygen Species and Urate Radicals

The EPR spectra were taken to identify the reactive oxygen radical (ROS) upon the possible activation of O_2_ or H_2_O_2_ by Cu-ZIF NMOFs, PAn-coated Cu-ZIF NMOFs, and UA-imprinted PAn-coated Cu-ZIF NMOFs, and BMPO was used as the trapping agent to trap both the superoxide radical (O_2_^–•^) and hydroxyl radical (^•^OH). Briefly, 50 μL of the mixture was prepared by mixing PBS (pH 7.4), BMPO, H_2_O_2_ (no addition in the case of the activation of O_2_), and catalysts in sequence with the final concentrations of 10 mM, 25 mM, 1 mM, and 50 μg mL^–1^, respectively, and was then loaded in glass capillary tube with an internal diameter of 1 mm. The EPR spectra of the mixture were recorded at a fixed time for each sample on an ELEXSYS 500 X-band spectrometer at RT. As the BMPO-O_2_^–•^ adduct is unstable and spontaneously decays into the BMPO-^•^OH adduct, the other set of experiments with the addition of superoxide dismutase (SOD) were conducted to distinguish the ROS following the same procedure. The effect of the UA on the EPR signal of the BMPO-O_2_^–•^ adduct upon the activation of O_2_ by UA-imprinted PAn-coated Cu-ZIF NMOFs was studied in the presence of variable concentrations of UA (25 μM, 125 μM, 250 μM, and 500 μM) with the same procedure. The concentrations of PBS (pH 7.4), BMPO, and UA-imprinted PAn-coated Cu-ZIF NMOFs were 10 mM, 25 mM, and 50 μg mL^–1^, respectively. To identify the urate radical, DEPMPO was used as the trapping reagent, and EPR spectra were obtained following the same procedure with the final concentration of PBS (pH 7.4), DEPMPO, UA (or allantoin; no addition in the control experiment), and UA-imprinted PAn-coated Cu-ZIF NMOFs to be 10 mM, 20 mM, 0.5 mM, and 50 μg mL^–1^, respectively.

The involvement of O_2_^–•^ in the aerobic oxidation of UA by UA-imprinted PAn-coated Cu-ZIF NMOFs was further explored by monitoring the activity of UA oxidation in the presence of SOD-depleting O_2_^–•^. Briefly, PBS (pH 7.4), UA, SOD, and the catalyst were mixed in sequence with the final concentrations of 10 mM, 75 μM, 0.1 units L^–1^ (or 0.2 units L^–1^), and 50 μg mL^–1^, respectively. Immediately, repeat-scan mode with a time interval of 10 min was started to record the time-dependent absorbance changes upon the catalyzed aerobic oxidation of UA using a UV-1900 UV–vis spectrophotometer at RT.

## Supporting Information Available

Additional SEM, HAADF-STEM, and the accompanying EDS element mapping images; PXRD, N_2_ adsorption–desorption (77 K) isotherms, pore-size distribution, and XPS spectra; table of *d*-spacing identified in the diffractogram; catalytic H_2_O_2_ and aerobic oxidation of UA; raw calorimetric titration curves and integrated curves; and EPR spectra (PDF)

## References

[ref1] Huang Y. Y.; Ren J. S.; Qu X. G. Nanozymes: Classification, catalytic mechanisms, activity regulation, and applications. Chem. Rev. 2019, 119 (6), 4357–4412. 10.1021/acs.chemrev.8b00672. 30801188

[ref2] Wu J. J. X.; Wang X. Y.; Wang Q.; Lou Z. P.; Li S. R.; Zhu Y. Y.; Qin L.; Wei H. Nanomaterials with enzyme-like characteristics (nanozymes): Next-generation artificial enzymes (II). Chem. Soc. Rev. 2019, 48 (4), 1004–1076. 10.1039/C8CS00457A. 30534770

[ref3] Wang D. D.; Jana D. L.; Zhao Y. L. Metal-organic framework derived nanozymes in biomedicine. Acc. Chem. Res. 2020, 53 (7), 1389–1400. 10.1021/acs.accounts.0c00268. 32597637

[ref4] Jin L. H.; Meng Z.; Zhang Y. Q.; Cai S. J.; Zhang Z. J.; Li C.; Shang L.; Shen Y. H. Ultrasmall Pt nanoclusters as robust peroxidase mimics for colorimetric detection of glucose in human serum. ACS Appl. Mater. Interfaces 2017, 9 (11), 10027–10033. 10.1021/acsami.7b01616. 28244734

[ref5] Ye H. H.; Liu Y. Z.; Chhabra A.; Lilla E.; Xia X. H. Polyvinylpyrrolidone (PVP)-capped Pt nanocubes with superior peroxidase-like activity. Chemnanomat 2017, 3 (1), 33–38. 10.1002/cnma.201600268.

[ref6] Shen X. M.; Liu W. Q.; Gao X. J.; Lu Z. H.; Wu X. C.; Gao X. F. Mechanisms of oxidase and superoxide dismutation-like activities of gold, silver, platinum, and palladium, and their alloys: A general way to the activation of molecular oxygen. J. Am. Chem. Soc. 2015, 137 (50), 15882–15891. 10.1021/jacs.5b10346. 26642084

[ref7] Liu Y. P.; Wang C. W.; Cai N.; Long S. H.; Yu F. Q. Negatively charged gold nanoparticles as an intrinsic peroxidase mimic and their applications in the oxidation of dopamine. J. Mater. Sci. 2014, 49 (20), 7143–7150. 10.1007/s10853-014-8422-x.

[ref8] Li Y. Y.; He X.; Yin J. J.; Ma Y. H.; Zhang P.; Li J. Y.; Ding Y. Y.; Zhang J.; Zhao Y. L.; Chai Z. F.; Zhang Z. Y. Acquired superoxide-scavenging ability of ceria nanoparticles. Angew. Chem., Int. Ed. 2015, 54 (6), 1832–1835. 10.1002/anie.201410398. 25515687

[ref9] Chen Y.; Liu Y.; Guo C. M.; Yin C.; Xie C.; Fan Q. L. Self-amplified competitive coordination of MnO_2_-doped CeO_2_ nanozyme for synchronously activated combination therapy. Adv. Funct. Mater. 2023, 33 (2), 2209927 10.1002/adfm.202209927.

[ref10] Ragg R.; Natalio F.; Tahir M. N.; Janssen H.; Kashyap A.; Strand D.; Strand S.; Tremel W. Molybdenum trioxide nanoparticles with intrinsic sulfite oxidase activity. ACS Nano 2014, 8 (5), 5182–5189. 10.1021/nn501235j. 24702461

[ref11] Natalio F.; André R.; Hartog A. F.; Stoll B.; Jochum K. P.; Wever R.; Tremel W. Vanadium pentoxide nanoparticles mimic vanadium haloperoxidases and thwart biofilm formation. Nat. Nanotechnol. 2012, 7 (8), 530–535. 10.1038/nnano.2012.91. 22751222

[ref12] Wang H.; Liu C. Q.; Liu Z.; Ren J. S.; Qu X. G. Specific oxygenated groups enriched graphene quantum dots as highly efficient enzyme mimics. Small 2018, 14 (13), 1703710 10.1002/smll.201703710. 29430831

[ref13] Wang S.; Cazelles R.; Liao W. C.; Vázquez-González M.; Zoabi A.; Abu-Reziq R.; Willner I. Mimicking horseradish peroxidase and NADH peroxidase by heterogeneous Cu^2+^-modified graphene oxide nanoparticles. Nano Lett. 2017, 17 (3), 2043–2048. 10.1021/acs.nanolett.7b00093. 28183178

[ref14] Vázquez-González M.; Liao W. C.; Cazelles R.; Wang S.; Yu X.; Gutkin V.; Willner I. Mimicking horseradish peroxidase functions using Cu^2+^-modified carbon nitride nanoparticles or Cu^2+^-modified carbon dots as heterogeneous catalysts. ACS Nano 2017, 11 (3), 3247–3253. 10.1021/acsnano.7b00352. 28234445

[ref15] Vázquez-González M.; Torrente-Rodríguez R. M.; Kozell A.; Liao W. C.; Cecconello A.; Campuzano S.; Pingarrón J. M.; Willner I. Mimicking peroxidase activities with prussian blue nanoparticles and their cyanometalate structural analogues. Nano Lett. 2017, 17 (8), 4958–4963. 10.1021/acs.nanolett.7b02102. 28656770

[ref16] Komkova M. A.; Karyakina E. E.; Karyakin A. A. Catalytically synthesized prussian blue nanoparticles defeating natural enzyme peroxidase. J. Am. Chem. Soc. 2018, 140 (36), 11302–11307. 10.1021/jacs.8b05223. 30118222

[ref17] Xiao J. Y.; Hai L.; Li Y. Y.; Li H.; Gong M. H.; Wang Z. F.; Tang Z. F.; Deng L.; He D. G. An ultrasmall Fe_3_O_4_-decorated polydopamine hybrid nanozyme enables continuous conversion of oxygen into toxic hydroxyl radical via GSH-depleted cascade redox reactions for intensive wound disinfection. Small 2022, 18 (9), 2105465 10.1002/smll.202105465. 34918449

[ref18] Liu Y. L.; Ai K. L.; Ji X. Y.; Askhatova D.; Du R.; Lu L. H.; Shi J. J. Comprehensive insights into the multi-antioxidative mechanisms of melanin nanoparticles and their application to protect brain from injury in ischemic stroke. J. Am. Chem. Soc. 2017, 139 (2), 856–862. 10.1021/jacs.6b11013. 27997170 PMC5752099

[ref19] Zheng L. M.; Wang F. Q.; Jiang C. R.; Ye S. J.; Tong J. Z.; Dramou P.; He H. Recent progress in the construction and applications of metal-organic frameworks and covalent-organic frameworks-based nanozymes. Coord. Chem. Rev. 2022, 471, 214760 10.1016/j.ccr.2022.214760.

[ref20] Li M. H.; Chen J. X.; Wu W. W.; Fang Y. X.; Dong S. J. Oxidase-like MOF-818 nanozyme with high specificity for catalysis of catechol oxidation. J. Am. Chem. Soc. 2020, 142 (36), 15569–15574. 10.1021/jacs.0c07273. 32790301

[ref21] Chen W. H.; Vázquez-González M.; Kozell A.; Cecconello A.; Willner I. Cu^2+^-modified metal-organic framework nanoparticles: A peroxidase-mimicking nanoenzyme. Small 2018, 14 (5), 1703149 10.1002/smll.201703149. 29205812

[ref22] Biniuri Y.; Albada B.; Wolff M.; Golub E.; Gelman D.; Willner I. Cu^2+^ or fe^3+^ terpyridine/aptamer conjugates: Nucleoapzymes catalyzing the oxidation of dopamine to aminochrome. ACS Catal. 2018, 8 (3), 1802–1809. 10.1021/acscatal.7b03454.

[ref23] Liu Z. W.; Wang F. M.; Ren J. S.; Qu X. G. A series of MOF/Ce-based nanozymes with dual enzyme-like activity disrupting biofilms and hindering recolonization of bacteria. Biomater 2019, 208, 21–31. 10.1016/j.biomaterials.2019.04.007. 30986610

[ref24] Chen W. H.; Vázquez-González M.; Zoabi A.; Abu-Reziq R.; Willner I. Biocatalytic cascades driven by enzymes encapsulated in metal-organic framework nanoparticles. Nat. Catal. 2018, 1 (9), 689–695. 10.1038/s41929-018-0117-2.

[ref25] Cai R.; Yang D.; Peng S. J.; Chen X. G.; Huang Y.; Liu Y.; Hou W. J.; Yang S. Y.; Liu Z. B.; Tan W. H. Single nanoparticle to 3D supercage: Framing for an artificial enzyme system. J. Am. Chem. Soc. 2015, 137 (43), 13957–13963. 10.1021/jacs.5b09337. 26464081 PMC4927331

[ref26] Gao L. Z.; Zhuang J.; Nie L.; Zhang J. B.; Zhang Y.; Gu N.; Wang T. H.; Feng J.; Yang D. L.; Perrett S.; Yan X. Intrinsic peroxidase-like activity of ferromagnetic nanoparticles. Nat. Nanotechnol. 2007, 2 (9), 577–583. 10.1038/nnano.2007.260. 18654371

[ref27] Bhattacharyya S.; Ali S. R.; Venkateswarulu M.; Howlader P.; Zangrando E.; De M.; Mukherjee P. S. Self-assembled Pd_12_ coordination cage as photoregulated oxidase-like nanozyme. J. Am. Chem. Soc. 2020, 142 (44), 18981–18989. 10.1021/jacs.0c09567. 33104330

[ref28] Chen J. X.; Ma Q.; Li M. H.; Chao D. Y.; Huang L.; Wu W. W.; Fang Y. X.; Dong S. J. Glucose-oxidase like catalytic mechanism of noble metal nanozymes. Nat. Commun. 2021, 12 (1), 3375 10.1038/s41467-021-23737-1. 34099730 PMC8184917

[ref29] Jiao M. Z.; Li Z. J.; Li X. L.; Zhang Z. J.; Yuan Q. P.; Vriesekoop F.; Liang H.; Liu J. W. Solving the H_2_O_2_ by-product problem using a catalase-mimicking nanozyme cascade to enhance glycolic acid oxidase. Chem. Eng. J. 2020, 388, 124249 10.1016/j.cej.2020.124249.

[ref30] Gao W. H.; He J. Y.; Chen L.; Meng X. Q.; Ma Y. N.; Cheng L. L.; Tu K. S.; Gao X. F.; Liu C.; Zhang M. Z.; Fan K. L.; Pang D. W.; Yan X. Y. Deciphering the catalytic mechanism of superoxide dismutase activity of carbon dot nanozyme. Nat. Commun. 2023, 14 (1), 160 10.1038/s41467-023-35828-2. 36631476 PMC9834297

[ref31] Li S. R.; Zhou Z. J.; Tie Z. X.; Wang B.; Ye M.; Du L.; Cui R.; Liu W.; Wan C. H.; Liu Q. Y.; Zhao S.; Wang Q.; Zhang Y. H.; Zhang S.; Zhang H. G.; Du Y.; Wei H. Data-informed discovery of hydrolytic nanozymes. Nat. Commun. 2022, 13 (1), 827 10.1038/s41467-022-28344-2. 35149676 PMC8837776

[ref32] Li F.; Li S.; Guo X. C.; Dong Y. H.; Yao C.; Liu Y. P.; Song Y. G.; Tan X. L.; Gao L. Z.; Yang D. Y. Chiral carbon dots mimicking topoisomerase I to mediate the topological rearrangement of supercoiled DNA enantioselectively. Angew. Chem., Int. Ed. 2020, 59 (27), 11087–11092. 10.1002/anie.202002904. 32212366

[ref33] Hong Q.; Yang H.; Fang Y. F.; Li W.; Zhu C. X.; Wang Z.; Liang S. C.; Cao X. W.; Zhou Z. X.; Shen Y. F.; Liu S. Q.; Zhang Y. J. Adaptable graphitic C_6_N_6_-based copper single-atom catalyst for intelligent biosensing. Nat. Commun. 2023, 14 (1), 2780 10.1038/s41467-023-38459-9. 37188673 PMC10185664

[ref34] Ouyang Y.; O’Hagan M. P.; Willner I. Functional catalytic nanoparticles (nanozymes) for sensing. Biosens. Bioelectron. 2022, 218, 114768 10.1016/j.bios.2022.114768. 36240630

[ref35] Ouyang Y.; Fadeev M.; Zhang P.; Carmieli R.; Sohn Y. S.; Karmi O.; Qin Y. L.; Chen X. H.; Nechushtai R.; Willner I. Aptamer-functionalized Ce^4+^-ion-modified C-Dots: Peroxidase mimicking aptananozymes for the oxidation of dopamine and cytotoxic effects toward cancer cells. ACS Appl. Mater. Interfaces 2022, 14 (50), 55365–55375. 10.1021/acsami.2c16199. 36475576 PMC9782376

[ref36] Jiang D. W.; Ni D. L.; Rosenkrans Z. T.; Huang P.; Yan X. Y.; Cai W. B. Nanozyme: New horizons for responsive biomedical applications. Chem. Soc. Rev. 2019, 48 (14), 3683–3704. 10.1039/C8CS00718G. 31119258 PMC6696937

[ref37] Zhang Y.; Wang Z. Y.; Li X. J.; Wang L.; Yin M.; Wang L. H.; Chen N.; Fan C. H.; Song H. Y. Dietary iron oxide nanoparticles delay aging and ameliorate neurodegeneration in drosophila. Adv. Mater. 2016, 28 (7), 1387–1393. 10.1002/adma.201503893. 26643597

[ref38] Xu B. L.; Wang H.; Wang W. W.; Gao L. Z.; Li S. S.; Pan X. T.; Wang H. Y.; Yang H. L.; Meng X. Q.; Wu Q. W.; Zheng L. R.; Chen S. M.; Shi X. H.; Fan K. L.; Yan X. Y.; Liu H. Y. A single-atom nanozyme for wound disinfection applications. Angew. Chem., Int. Ed. 2019, 58 (15), 4911–4916. 10.1002/anie.201813994. 30697885

[ref39] Li Y. Y.; Yu P.; Wen J.; Sun H.; Wang D. Q.; Liu J. M.; Li J. S.; Chu H. T. Nanozyme-based stretchable hydrogel of low hysteresis with antibacterial and antioxidant dual functions for closely fitting and wound healing in movable parts. Adv. Funct. Mater. 2022, 32 (13), 2110720 10.1002/adfm.202110720.

[ref40] Pan M. M.; Ouyang Y.; Song Y. L.; Si L. Q.; Jiang M.; Yu X.; Xu L.; Willner I. Au^3+^-functionalized UiO-67 metal-organic framework nanoparticles: O_2_^•-^ and ^•^OH generating nanozymes and their antibacterial functions. Small 2022, 18 (23), 2200548 10.1002/smll.202200548. 35460191

[ref41] Wu W. W.; Huang L.; Wang E. K.; Dong S. J. Atomic engineering of single-atom nanozymes for enzyme-like catalysis. Chem. Sci. 2020, 11 (36), 9741–9756. 10.1039/D0SC03522J. 34094238 PMC8162425

[ref42] Koyappayil A.; Kim H. T.; Lee M. H. Laccase-like’ properties of coral-like silver citrate micro-structures for the degradation and determination of phenolic pollutants and adrenaline. J. Hazard. Mater. 2021, 412, 125211 10.1016/j.jhazmat.2021.125211. 33516111

[ref43] Zhang X. L.; Li G. L.; Chen G.; Wu D.; Wu Y. N.; James T. D. Enzyme mimics for engineered biomimetic cascade nanoreactors: Mechanism, applications, and prospects. Adv. Funct. Mater. 2021, 31 (50), 2106139 10.1002/adfm.202106139.

[ref44] Ouyang Y.; Biniuri Y.; Fadeev M.; Zhang P.; Carmieli R.; Vazquez-Gonzalez M.; Willner I. Aptamer-modified Cu^2+^-functionalized C-Dots: Versatile means to improve nanozyme activities-″aptananozymes. J. Am. Chem. Soc. 2021, 143 (30), 11510–11519. 10.1021/jacs.1c03939. 34286967 PMC8856595

[ref45] Ouyang Y.; Fadeev M.; Zhang P.; Carmieli R.; Li J.; Sohn Y. S.; Karmi O.; Nechushtai R.; Pikarsky E.; Fan C. H.; Willner I. Aptamer-modified Au nanoparticles: Functional nanozyme bioreactors for cascaded catalysis and catalysts for chemodynamic treatment of cancer cells. ACS Nano 2022, 16 (11), 18232–18243. 10.1021/acsnano.2c05710. 36286233 PMC9706657

[ref46] Zhang Z. J.; Zhang X. H.; Liu B. W.; Liu J. W. Molecular imprinting on inorganic nanozymes for hundred-fold enzyme specificity. J. Am. Chem. Soc. 2017, 139 (15), 5412–5419. 10.1021/jacs.7b00601. 28345903

[ref47] Wulff G. Enzyme-like catalysis by molecularly imprinted polymers. Chem. Rev. 2002, 102 (1), 1–27. 10.1021/cr980039a. 11782127

[ref48] Whitcombe M. J.; Vulfson E. N. Imprinted polymers. Adv. Mater. 2001, 13 (7), 467–478. 10.1002/1521-4095(200104)13:7<467::AID-ADMA467>3.0.CO;2-T.

[ref49] Song Z. H.; Li J. H.; Lu W. H.; Li B. W.; Yang G. Q.; Bi Y.; Arabi M.; Wang X. Y.; Ma J. P.; Chen L. X. Molecularly imprinted polymers based materials and their applications in chromatographic and electrophoretic separations. TrAC, Trends Anal. Chem. 2022, 146, 116504 10.1016/j.trac.2021.116504.

[ref50] Yoshikawa M.; Tharpa K.; Dima S. O. Molecularly imprinted membranes: Past, present, and future. Chem. Rev. 2016, 116 (19), 11500–11528. 10.1021/acs.chemrev.6b00098. 27610706

[ref51] Wulff G.; Liu J. Q. Design of biomimetic catalysts by molecular imprinting in synthetic polymers: The role of transition state stabilization. Acc. Chem. Res. 2012, 45 (2), 239–247. 10.1021/ar200146m. 21967389

[ref52] Ye L.; Mosbach K. Molecular imprinting: Synthetic materials as substitutes for biological antibodies and receptors. Chem. Mater. 2008, 20 (3), 859–868. 10.1021/cm703190w.

[ref53] Hu X. B.; Li G. T.; Huang J.; Zhang D.; Qiu Y. Construction of self-reporting specific chemical sensors with high sensitivity. Adv. Mater. 2007, 19 (24), 4327–4332. 10.1002/adma.200701084.

[ref54] Sallacan N.; Zayats M.; Bourenko T.; Kharitonov A. B.; Willner I. Imprinting of nucleotide and monosaccharide recognition sites in acrylamidephenylboronic acid-acrylamide copolymer membranes associated with electronic transducers. Anal. Chem. 2002, 74 (3), 702–712. 10.1021/ac0109873. 11838699

[ref55] Elsabahy M.; Wooley K. L. Design of polymeric nanoparticles for biomedical delivery applications. Chem. Soc. Rev. 2012, 41 (7), 2545–2561. 10.1039/c2cs15327k. 22334259 PMC3299918

[ref56] Cunliffe D.; Kirby A.; Alexander C. Molecularly imprinted drug delivery systems. Adv. Drug Delivery Rev. 2005, 57 (12), 1836–1853. 10.1016/j.addr.2005.07.015. 16226341

[ref57] Ploetz E.; Engelke H.; Lächelt U.; Wuttke S. The chemistry of reticular framework nanoparticles: MOF, ZIF, and COF materials. Adv. Funct. Mater. 2020, 30 (41), 1909062 10.1002/adfm.201909062.

[ref58] Navarro Poupard M. F.; Polo E.; Taboada P.; Arenas-Vivo A.; Horcajada P.; Pelaz B.; del Pino P. Aqueous synthesis of Copper(II)-imidazolate nanoparticles. Inorg. Chem. 2018, 57 (19), 12056–12065. 10.1021/acs.inorgchem.8b01612. 30221514

[ref59] Kaneti Y. V.; Dutta S.; Hossain M. S. A.; Shiddiky M. J. A.; Tung K. L.; Shieh F. K.; Tsung C. K.; Wu K. C. W.; Yamauchi Y. Strategies for improving the functionality of zeolitic imidazolate frameworks: Tailoring nanoarchitectures for functional applications. Adv. Mater. 2017, 29 (38), 1700213 10.1002/adma.201700213. 28833624

[ref60] Li C.; Ye J.; Yang X.; Liu S.; Zhang Z.; Wang J.; Zhang K.; Xu J.; Fu Y.; Yang P. Fe/mn bimetal-doped ZIF-8-coated luminescent nanoparticles with up/downconversion dual-mode emission for tumor self-enhanced nir-II imaging and catalytic therapy. ACS Nano 2022, 16 (11), 18143–18156. 10.1021/acsnano.2c05152. 36260703

[ref61] Wang C.; Tuninetti J.; Wang Z.; Zhang C.; Ciganda R.; Salmon L.; Moya S.; Ruiz J.; Astruc D. Hydrolysis of ammonia-borane over ni/ZIF-8 nanocatalyst: High efficiency, mechanism, and controlled hydrogen release. J. Am. Chem. Soc. 2017, 139 (33), 11610–11615. 10.1021/jacs.7b06859. 28763209

[ref62] Li J. X.; Wang J. L.; Chai T. Q.; Yang F. Q. One-pot synthesized copper-imidazole-2-carboxaldehyde complex material with oxidase-like activity for the colorimetric detection of glutathione and ascorbic acid. Heliyon 2023, 9 (11), e22099 10.1016/j.heliyon.2023.e22099. 38027898 PMC10663933

[ref63] Cho K. Y.; An H.; Do X. H.; Choi K.; Yoon H. G.; Jeong H.-K.; Lee J. S.; Baek K.-Y. Synthesis of amine-functionalized ZIF-8 with 3-amino-1,2,4-triazole by postsynthetic modification for efficient co_2_-selective adsorbents and beyond. J. Mater. Chem. A 2018, 6 (39), 18912–18919. 10.1039/C8TA02797H.

[ref64] Soleimani B.; Niknam Shahrak M.; Walton K. S. The influence of different functional groups on enhancing CO_2_ capture in metal-organic framework adsorbents. J. Taiwan Inst. Chem. Eng. 2024, 163, 105638 10.1016/j.jtice.2024.105638.

[ref65] Xi B. J.; Tan Y. C.; Zeng H. C. A general synthetic approach for integrated nanocatalysts of Metal-Silica@ZIFs. Chem. Mater. 2016, 28 (1), 326–336. 10.1021/acs.chemmater.5b04147.

[ref66] Chen W. H.; Luo G. F.; Vázquez-González M.; Cazelles R.; Sohn Y. S.; Nechushtai R.; Mandel Y.; Willner I. Glucose-responsive metal-organic-framework nanoparticles act as ″smart″ sense-and-treat carriers. ACS Nano 2018, 12 (8), 7538–7545. 10.1021/acsnano.8b03417. 29969227

[ref67] Zhou Z. X.; Vázquez-González M.; Willner I. Stimuli-responsive metal-organic framework nanoparticles for controlled drug delivery and medical applications. Chem. Soc. Rev. 2021, 50 (7), 4541–4563. 10.1039/D0CS01030H. 33625421

[ref68] Yang J.; Zhang F. J.; Lu H. Y.; Hong X.; Jiang H. L.; Wu Y.; Li Y. D. Hollow Zn/Co ZIF particles derived from core-shell ZIF-67@ZIF-8 as selective catalyst for the semi-hydrogenation of acetylene. Angew. Chem., Int. Ed. 2015, 54 (37), 10889–10893. 10.1002/anie.201504242. 26333054

[ref69] Chen C. C.; Vázquez-González M.; O’Hagan M. P.; Ouyang Y.; Wang Z. H.; Willner I. Enzyme-loaded hemin/g-quadruplex-modified ZIF-90 metal-organic framework nanoparticles: Bioreactor nanozymes for the cascaded oxidation of N-hydroxy-L-arginine and sensing applications. Small 2022, 18 (11), 2104420 10.1002/smll.202104420. 35037383

[ref70] Wu Y.; Qin Y. L.; Muppidathi M.; Carmieli R.; Fadeev M.; Lei W.; Xia M. Z.; Willner I. Functional nanozymes consisting of Co^2+^-ZIF-67 metal-organic framework nanoparticles and Co^2+^-ZIF-67/polyaniline conjugates. Adv. Funct. Mater. 2023, 34 (3), 2306929 10.1002/adfm.202306929.

[ref71] Rees F.; Hui M.; Doherty M. Optimizing current treatment of gout. Nat. Rev. Rheumatol. 2014, 10 (5), 271–283. 10.1038/nrrheum.2014.32. 24614592

[ref72] Dehlin M.; Jacobsson L.; Roddy E. Global epidemiology of gout: Prevalence, incidence, treatment patterns and risk factors. Nat. Rev. Rheumatol. 2020, 16 (7), 380–390. 10.1038/s41584-020-0441-1. 32541923

[ref73] Kuo C. F.; Grainge M. J.; Zhang W. Y.; Doherty M. Global epidemiology of gout: Prevalence, incidence and risk factors. Nat. Rev. Rheumatol. 2015, 11 (11), 649–662. 10.1038/nrrheum.2015.91. 26150127

[ref74] Dalbeth N.; Merriman T. R.; Stamp L. K. Gout. Lancet 2016, 388 (10055), 2039–2052. 10.1016/S0140-6736(16)00346-9. 27112094

[ref75] Zhang Y.; Hou C. J.; Zhao P.; Zeng X.; Liu Y. Y.; Chen J.; Gao Y. F.; Wang C. C.; Hou J. Z.; Huo D. Q. Fe single-atom nanozyme-modified wearable hydrogel patch for precise analysis of uric acid at rest. ACS Appl. Mater. Interfaces 2023, 15 (37), 43541–43549. 10.1021/acsami.3c08978. 37694575

[ref76] Dong Y. Q.; Chi Y. W.; Lin X. M.; Zheng L. Y.; Chen L. C.; Chen G. N. Nano-sized platinum as a mimic of uricase catalyzing the oxidative degradation of uric acid. Phys. Chem. Chem. Phys. 2011, 13 (13), 6319–6324. 10.1039/c0cp01759k. 21359394

[ref77] Schlesinger N.; Pérez-Ruiz F.; Lioté F. Mechanisms and rationale for uricase use in patients with gout. Nat. Rev. Rheumatol. 2023, 19 (10), 640–649. 10.1038/s41584-023-01006-3. 37684360

[ref78] Lin A. Q.; Sun Z. Y.; Xu X. Q.; Zhao S.; Li J. W.; Sun H.; Wang Q.; Jiang Q.; Wei H.; Shi D. Q. Self-cascade uricase/catalase mimics alleviate acute gout. Nano Lett. 2022, 22 (1), 508–516. 10.1021/acs.nanolett.1c04454. 34968071

[ref79] Xi J. Q.; Zhang R. F.; Wang L. M.; Xu W.; Liang Q.; Li J. Y.; Jiang J.; Yang Y. L.; Yan X. Y.; Fan K. L.; Gao L. Z. A nanozyme-based artificial peroxisome ameliorates hyperuricemia and ischemic stroke. Adv. Funct. Mater. 2021, 31 (9), 2007130 10.1002/adfm.202007130.

[ref80] Liu W.; Kumar J.; Tripathy S.; Senecal K. J.; Samuelson L. Enzymatically synthesized conducting polyaniline. J. Am. Chem. Soc. 1999, 121 (1), 71–78. 10.1021/ja982270b.

[ref81] Kahn K.; Serfozo P.; Tipton P. A. Identification of the true product of the urate oxidase reaction. J. Am. Chem. Soc. 1997, 119 (23), 5435–5442. 10.1021/ja970375t.

[ref82] Bui S.; von Stetten D.; Jambrina P. G.; Prangé T.; Colloc’h N.; de Sanctis D.; Royant A.; Rosta E.; Steiner R. A. Direct evidence for a peroxide intermediate and a reactive enzyme-substrate-dioxygen configuration in a cofactor-free oxidase. Angew. Chem., Int. Ed. 2014, 53 (50), 13710–13714. 10.1002/anie.201405485. PMC4502973 25314114

[ref83] Wei D. H.; Huang X. Q.; Qiao Y.; Rao J. J.; Wang L.; Liao F.; Zhan C. G. Catalytic mechanisms for cofactor-free oxidase-catalyzed reactions: Reaction pathways of uricase-catalyzed oxidation and hydration of uric acid. ACS Catal. 2017, 7 (7), 4623–4636. 10.1021/acscatal.7b00901. 28890842 PMC5589204

[ref84] Wang K. Y.; Hong Q.; Zhu C. X.; Xu Y.; Li W.; Wang Y.; Chen W. H.; Gu X.; Chen X. H.; Fang Y. F.; Shen Y. F.; Liu S. Q.; Zhang Y. J. Metal-ligand dual-site single-atom nanozyme mimicking urate oxidase with high substrates specificity. Nat. Commun. 2024, 15 (1), 5705 10.1038/s41467-024-50123-4. 38977710 PMC11231224

[ref85] Xi Z.; Xie J.; Hu J.; Wang Q.-C.; Wang Z.; Yang X.; Zong L.; Zhang M.; Sun X.; Sun S.; Han J. Polyvinylpyrrolidone-coated cubic hollow nanocages of PdPt_3_ and PdIr_3_ as highly efficient self-cascade uricase/peroxidase mimics. Nano Lett. 2024, 24 (11), 3432–3440. 10.1021/acs.nanolett.4c00071. 38391135

[ref86] Luo Y.; Orban M.; Kustin K.; Epstein I. R. Mechanistic study of oscillations and bistability in the Cu(II)-catalyzed reaction between H_2_O_2_ and KSCN. J. Am. Chem. Soc. 1989, 111 (13), 4541–4548. 10.1021/ja00195a001.

