# Supplementary material for: Molecularly Imprinted Polyaniline-Coated Cu-Zeolitic Imidazolate Framework Nanoparticles: Uricase-Mimicking “Polynanozyme” Catalyzing Uric Acid Oxidation
Source: ACS Nano. 2025 Mar 5;19(10):9981–93. doi: 10.1021/acsnano.4c16272 (PMC11924329; doi:10.1021/acsnano.4c16272)
Supplement: Supplementary file 1 — nn4c16272_si_001.pdf [file nn4c16272_si_001.pdf]

Supporting information for

# Molecularly Imprinted Polyaniline-Coated Cu-Zeolitic Imidazolate Framework Nanoparticles: Uricase-Mimicking "Polynanozyme" Catalyzing Uric Acid Oxidation

Xinghua Chen[1], Yi Wu[1,4], Yunlong Qin[1], Raanan Carmieli[2], Inna Popov[3], Vitaly Gutkin[3], Chunhai Fan[5] and Itamar Willner[1*]

[1]Institute of Chemistry, The Hebrew University of Jerusalem, Jerusalem 91904, Israel

[2]Department of Chemical Research Support, Weizmann Institute of Science, Rehovot 76100, Israel

[3]The Center for Nanoscience and Nanotechnology, The Hebrew University of Jerusalem, Jerusalem 91904, Israel

[4]School of Chemistry and Chemical Engineering, Nanjing University of Science and Technology, Nanjing 210094, China

[5]School of Chemistry and Chemical Engineering, Frontiers Science Center for Transformative Molecules, National Center for Translational Medicine, Shanghai Jiao Tong University, Shanghai 200240, China

*E-mail: willnea@vms.huji.ac.il

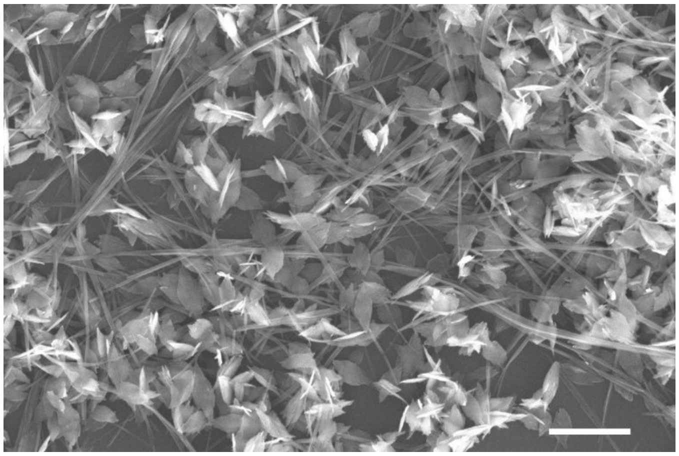

**Figure S1.** SEM image of Cu-ZIF NMOFs. Scale bar, 1 μm.

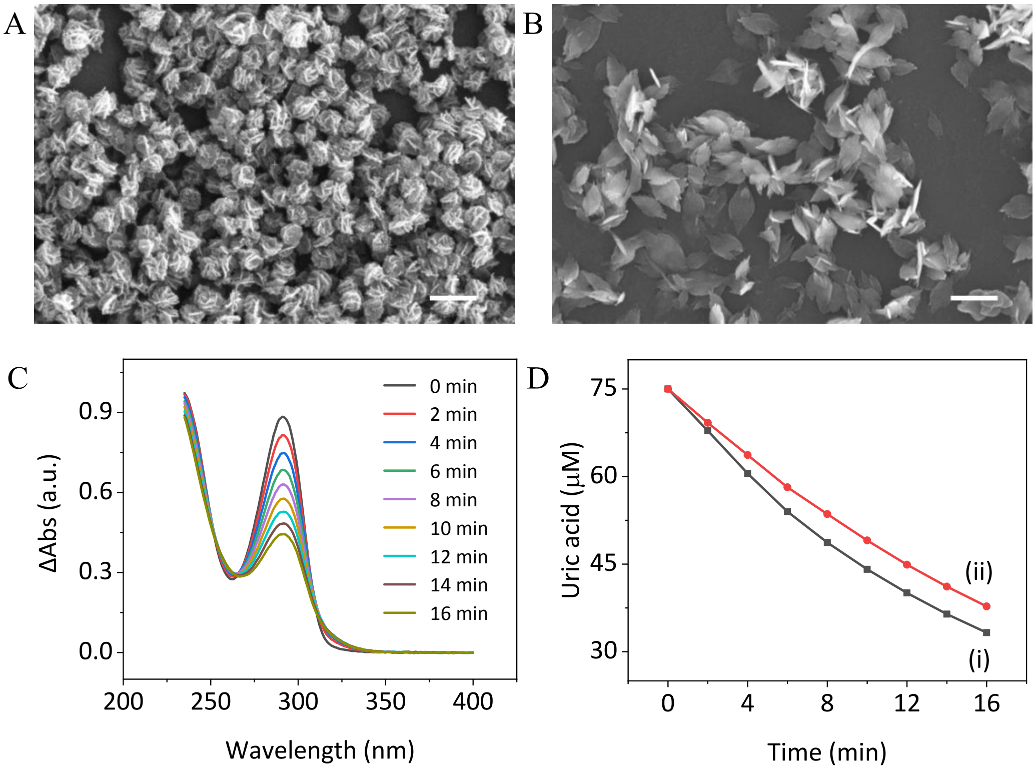


**Figure S2.** SEM image of Cu-ZIF NMOFs treated with acetone (A) and with acetone and water in sequence (B), scale bar, 0.5 μm. (C) Time-dependent absorbance changes of UA upon the acetone-treated Cu-ZIF NMOFs catalyzed oxidation of UA by $H_2O_2$. Time-dependent concentration changes of UA upon the catalyzed oxidation of UA by $H_2O_2$: i, water-treated Cu-ZIF NMOFs; ii, Cu-ZIF NMOFs treated with acetone and water in sequence. In all experiments, UA, 75 μM, $H_2O_2$, 5 mM and catalyst, 50 μg $mL^{-1}$, were used.

The mixture of solid flakes and fibers suspended in acetone resulted in spherical aggregates, ca. 250 nm diameter, **Figure S2A**. Resuspension of the solid spherical NMOFs formed in acetone with water resulted in the flake structure without the fibers, **Figure S2B**. The catalytic function of the mixture of flake and fibers of the Cu-ZIF NMOFs that will be described throughout the study are, however, very similar to that of the flakes, lacking the fibers, generated by a water-acetone-water cyclic treatment, **Figure S2C**,**2D**. (The flakes/spherical Cu-ZIF NMOFs transitions in water/acetone are reversible.)

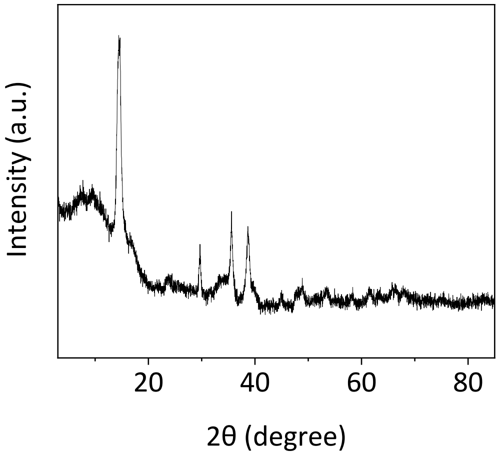


**Figure S3.** PXRD spectrum of Cu-ZIF NMOFs.

A

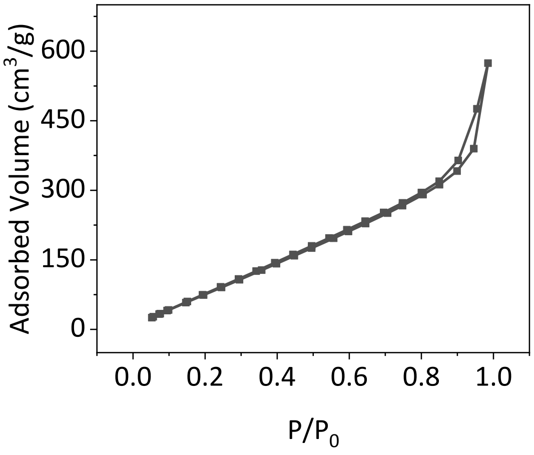


B

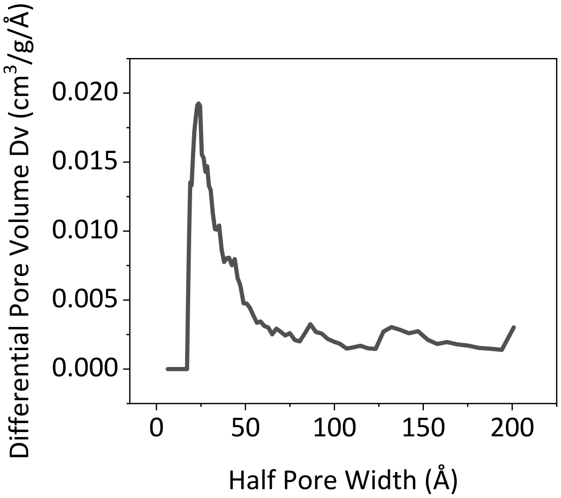


**Figure S4.** $N_2$ adsorption-desorption (77 K) isotherms (A) and pore-size distribution (calculated by using a slit/cylinder NLDFT equilibrium model) of Cu-ZIF NMOFs.

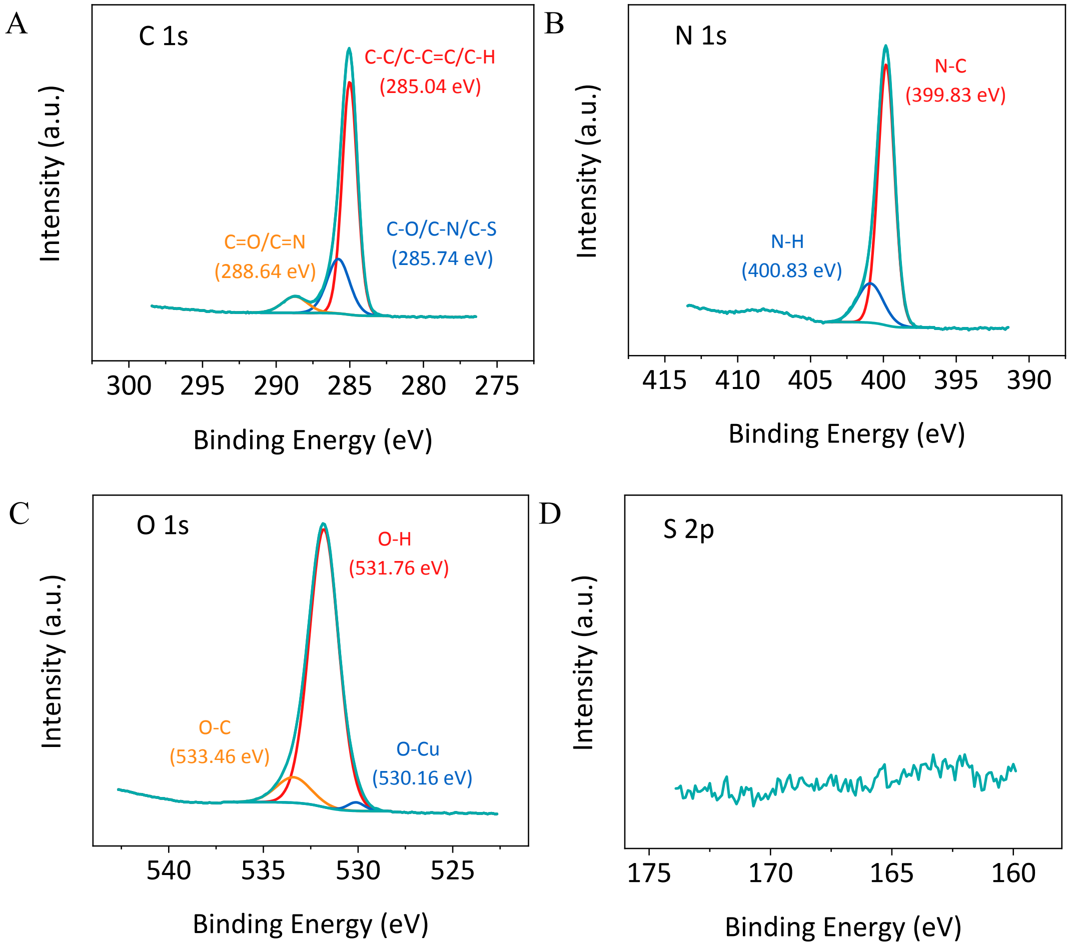


**Figure S5.** Deconvoluted C 1s (A), N 1s (B), O 1s (C) and S 2p (D) XPS spectra of Cu-ZIF NMOFs.

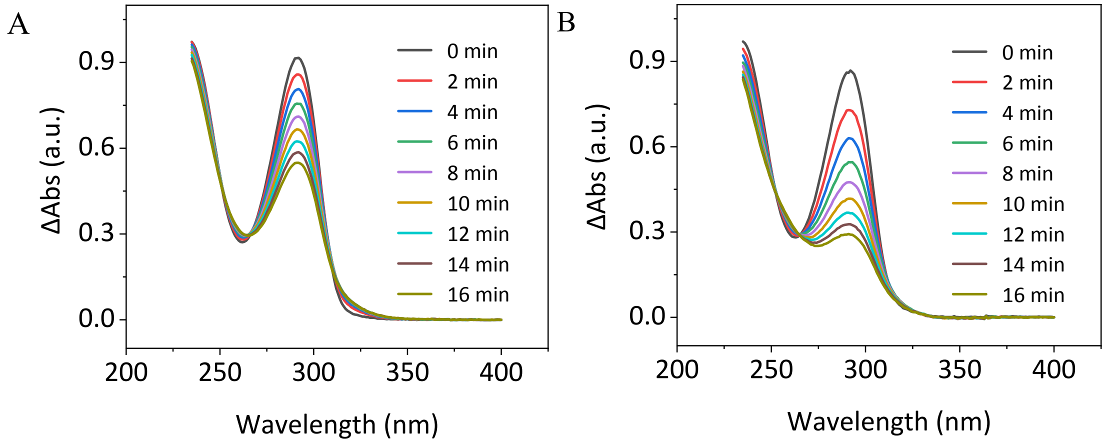


**Figure S6.** Absorbance spectra changes upon catalyzed $H_2O_2$ oxidation of UA in the presence of different concentrations of Cu-ZIF NMOFs: **A**, 25 μg $mL^{-1}$; **B**, 75 μg $mL^{-1}$. In all experiments, UA, 75 μM, and $H_2O_2$, 5 mM, were used.

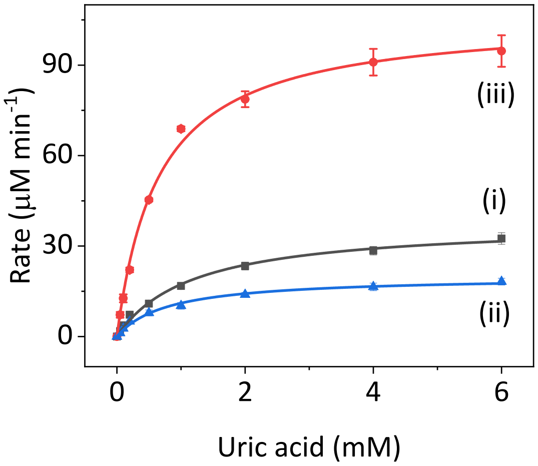


**Figure S7.** Rates of catalytic oxidation of UA with $H_2O_2$, 5 mM, at variable concentrations of UA by: **i**, Cu-ZIF NMOFs, 50 μg $mL^{-1}$; **ii**, PAn-coated Cu-ZIF NMOFs, 50 μg $mL^{-1}$; **iii**, UA-imprinted PAn-coated Cu-ZIF NMOFs, 50 μg $mL^{-1}$. The $V_{max}$ and $K_M$ values of the PAn-coated Cu-ZIF NMOFs are 20 μM $min^{-1}$ and 0.8 mM, respectively. The $V_{max}$ and $K_M$ values of the UA-imprinted PAn-coated Cu-ZIF NMOFs are 106 μM $min^{-1}$ and 0.6 mM, respectively.

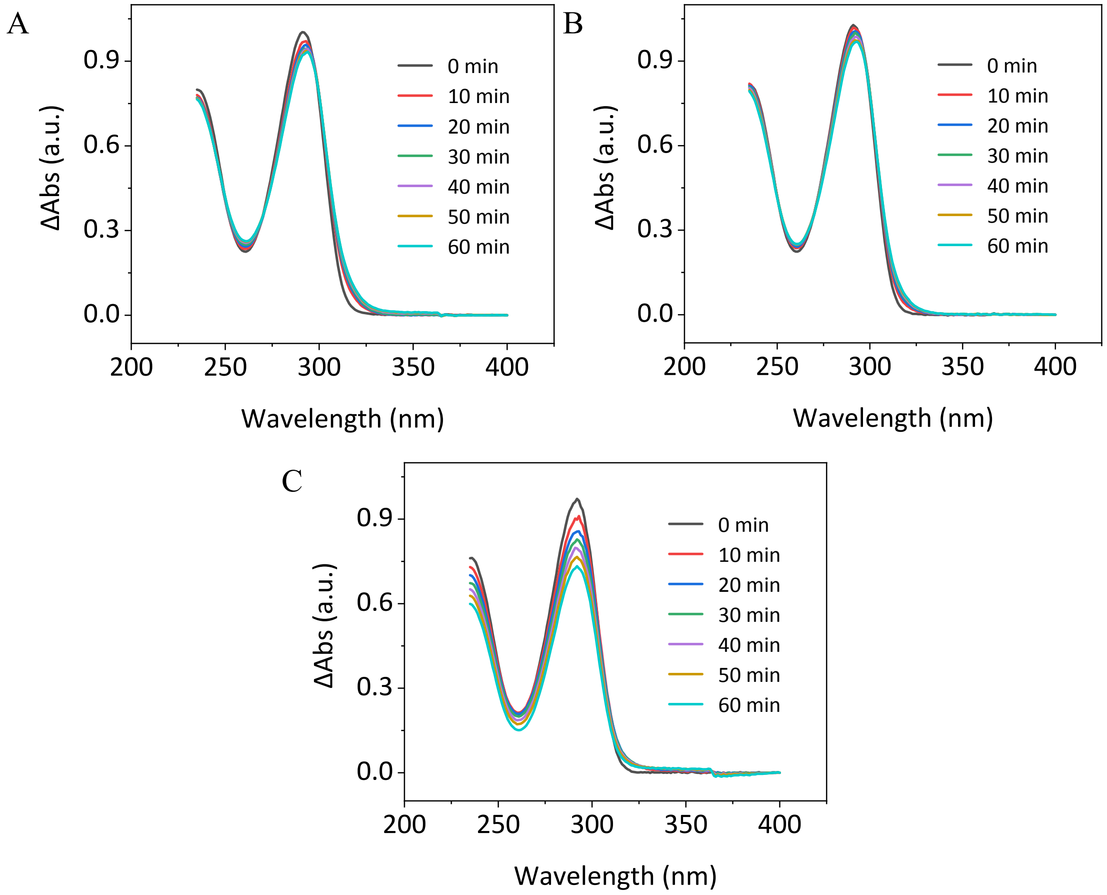


**Figure S8.** Absorbance spectra changes upon catalyzed aerobic oxidation of UA, 75 μM, by Cu-ZIF NMOFs, 50 μg $mL^{-1}$ (**A**), PAn-coated Cu-ZIF NMOFs, 50 μg $mL^{-1}$ (**B**), and UA-imprinted PAn-coated Cu-ZIF NMOFs, 50 μg $mL^{-1}$ (**C**).

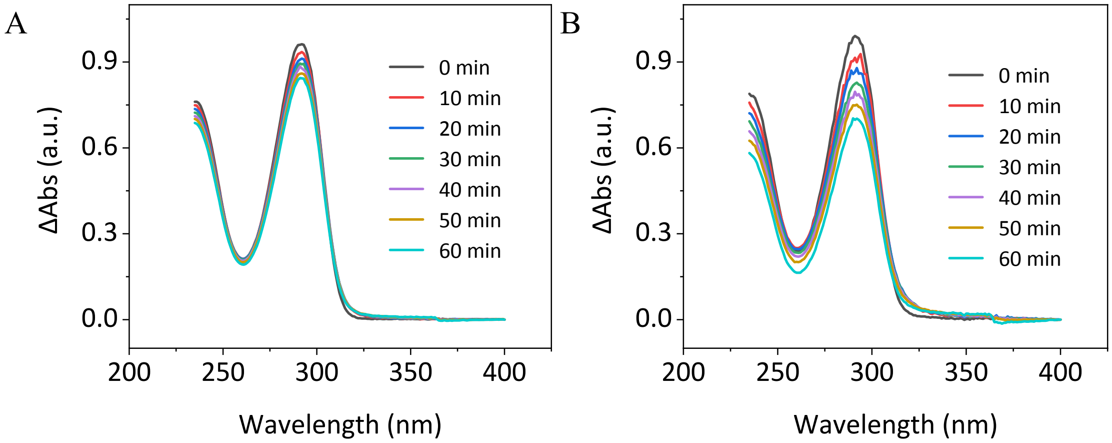


**Figure S9.** Absorbance spectra changes upon catalyzed aerobic oxidation of UA, 75 μM, in the presence of variable concentration of UA-imprinted PAn-coated Cu-ZIF NMOFs: **A**, 25 μg $mL^{-1}$; **B**, 75 μg $mL^{-1}$.

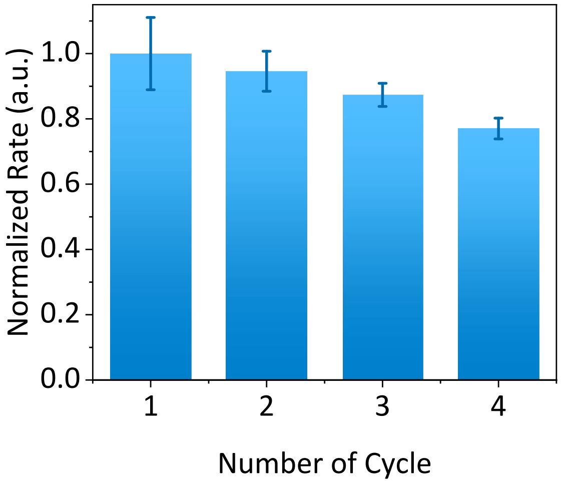


**Figure S10**. The normalized rates of recycling aerobic oxidation of UA, 75 µM, catalyzed by UA-imprinted PAn-coated Cu-ZIF NMOFs, 50 µg $mL^{-1}$.

The catalytic stability of the oxidase properties of the imprinted PAn-coated Cu-ZIF NMOFs was examined. The particles after a reaction cycle were precipitated and the catalytic oxidase functions of the resulting particles were examined using repeated cycles. The results are presented in **Figure S10**. Gradual loss in the catalytic oxidase functions of the NMOFs is observed (ca. 20 % less after the forth cycle). This activity loss is attributed to weight loss of the particles associated with the recycling of the catalyst.

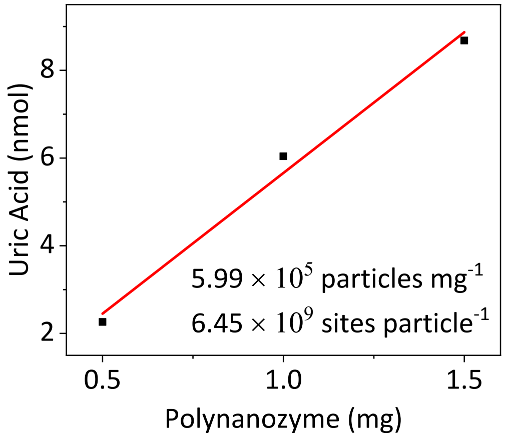


**Figure S11.** The average number of imprinted sites associated with a UA-imprinted PAn-coated particle.

The particle number of UA-imprinted PAn-coated Cu-ZIF NMOFs was determined using a TC20 automated cell counter. To estimate the average number of imprinted sites associated with the imprinted PAn coating of a single particle, 50 μM UA was incubated with variable concentrations of particles (0.5 mg, 1 mg and 1.5 mg particles in 200 μL of solution) at $N_2$ condition for 5 min. Subsequently, gentle centrifugation was performed, and the absorbance of the supernatant was measured to calculate the concentration of the residual UA. The number of the imprinted sites of a single particle was estimated by calculating the number of the UA associating to the imprinted sites of the particle.

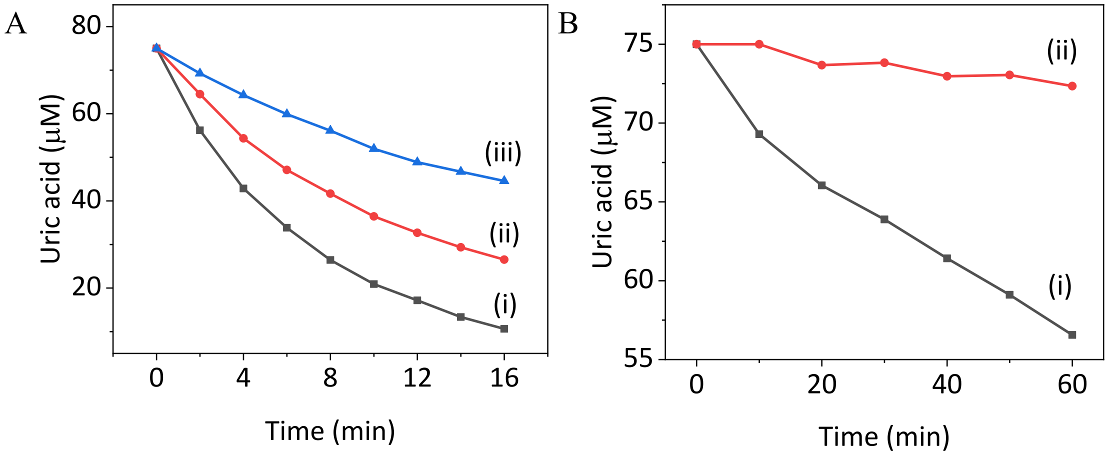


**Figure S12. (A)** Time-dependent concentration changes of UA upon catalytic $H_2O_2$ oxidation of UA, 75 µM, to allantoin by UA-imprinted PAn-coated Cu-ZIF NMOFs in the absence (**i**) or presence of different concentration of hypoxanthine: **ii,** 75 µM and **iii,** 375 µM. **(B)** Time-dependent concentration changes of UA upon catalytic aerobic oxidation of UA, 75 µM, to allantoin by UA-imprinted PAn-coated Cu-ZIF NMOFs in the absence (**i**) or presence (**ii**) of 375 µM hypoxanthine.

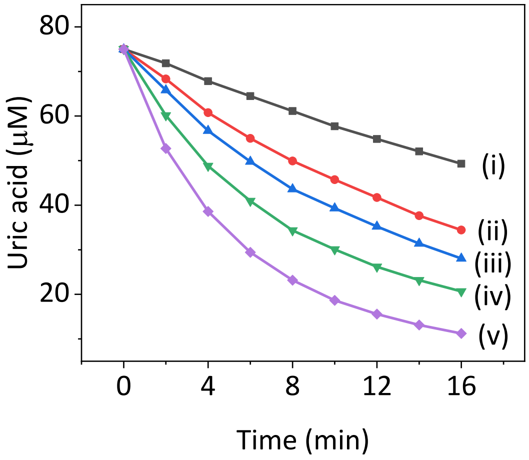


**Figure S13.** Time-dependent concentration changes of UA upon catalytic $H_2O_2$ oxidation of UA, 75 μM, to allantoin by UA-imprinted PAn-coated Cu-ZIF NMOFs obtained in the absence (**i**) or presence of variable concentrations of UA during imprinting process: **ii,** 2 mM; **iii**, 4 mM; **iv**, 6 mM; **v**, 8 mM. Note: the UA-imprinted PAn-coated Cu-ZIF discussed throughout the paper was synthesized with 8 mM UA unless otherwise specified.

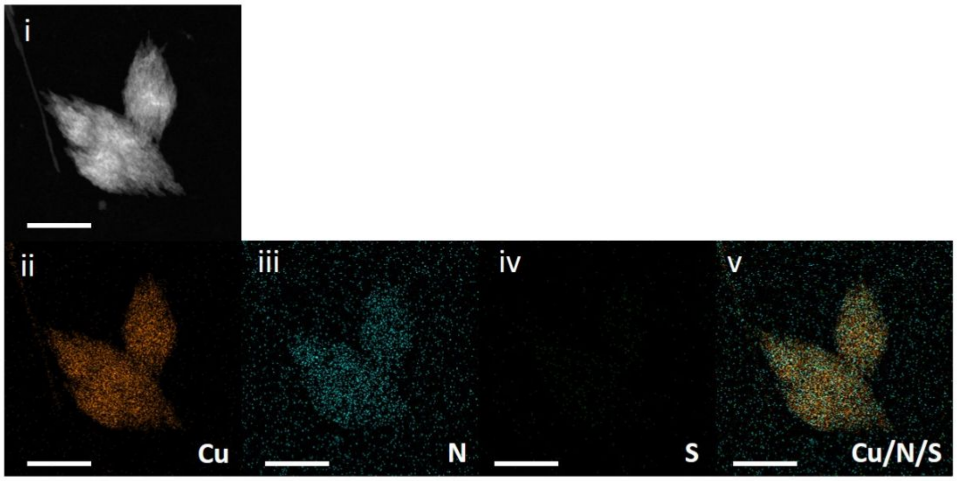


**Figure S14.** HAADF-STEM image (**i**) and the accompanying EDS element mapping images of Cu-ZIF NMOFs: **ii**, Cu; **iii**, N; **iv**, S; **v**, overlay of **ii**-**iv**. Scale bar, 200 nm.

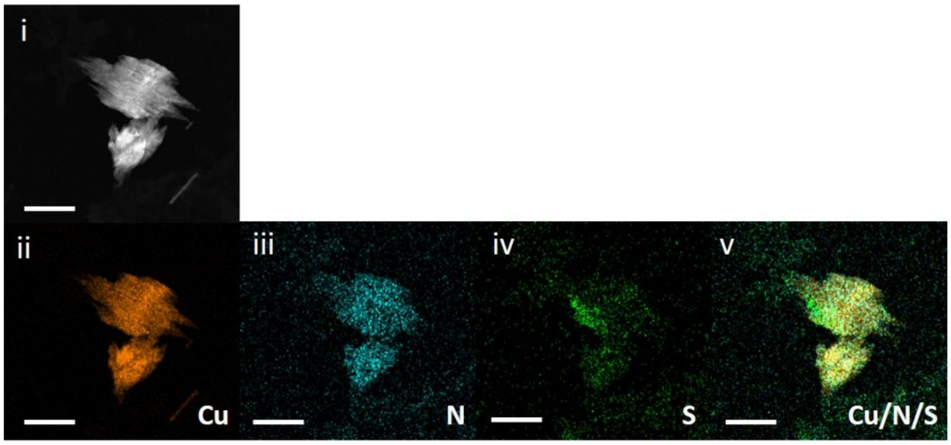


**Figure S15.** HAADF-STEM image (**i**) and the accompanying EDS element mapping images of the PAn-coated Cu-ZIF NMOFs: **ii**, Cu; **iii**, N; **iv**, S; **v**, overlay of **ii-iv**. Scale bar, 200 nm.

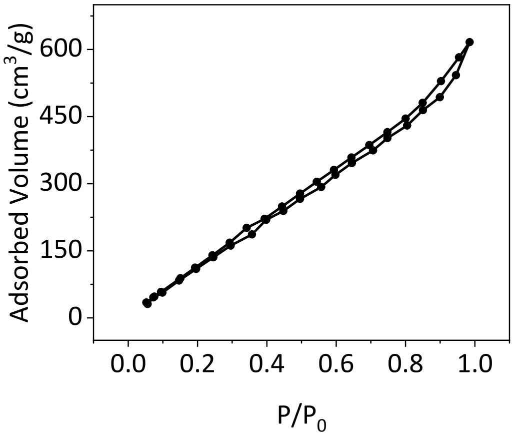


**Figure S16.** $N_2$ adsorption-desorption (77 K) isotherms of UA-imprinted PAn-coated Cu-ZIF NMOFs.

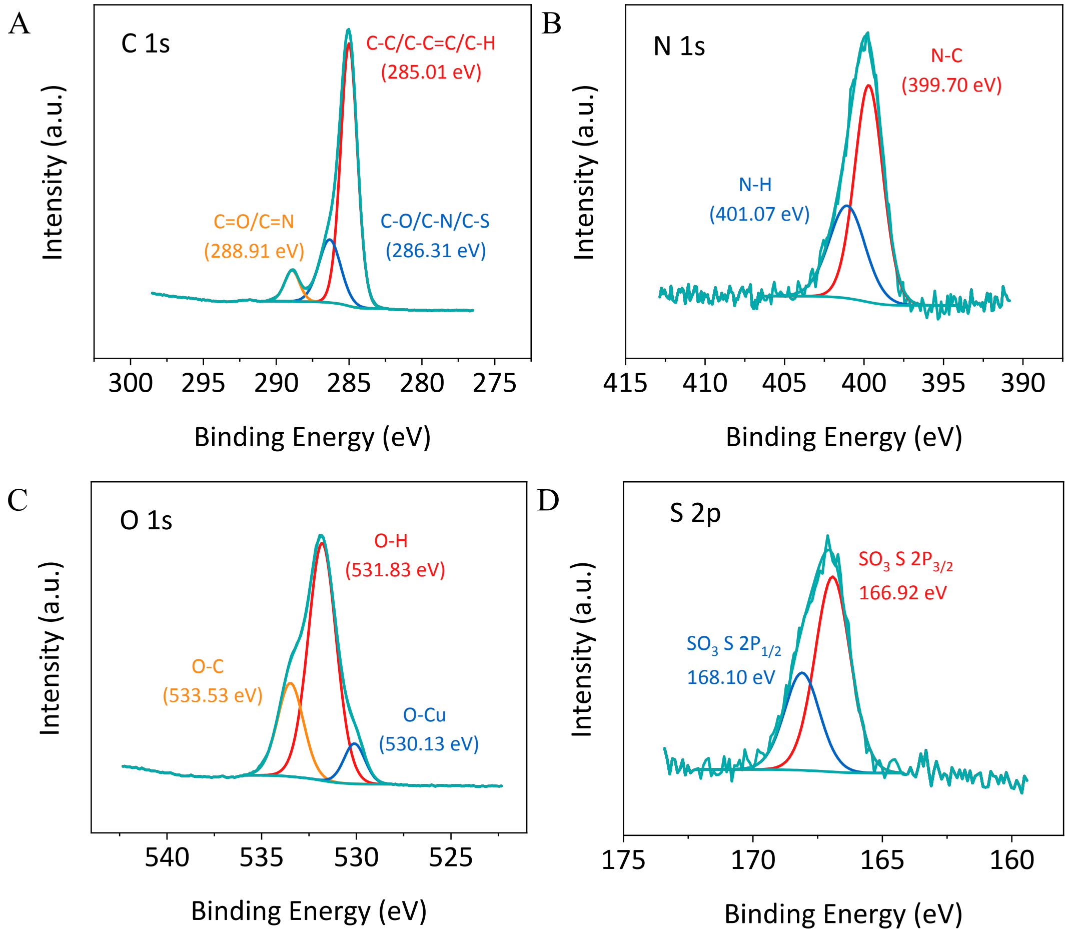


**Figure S17.** Deconvoluted C 1s (**A**), N 1s (**B**), O 1s (**C**) and S 2p (**D**) XPS spectra of PAn-coated Cu-ZIF NMOFs.

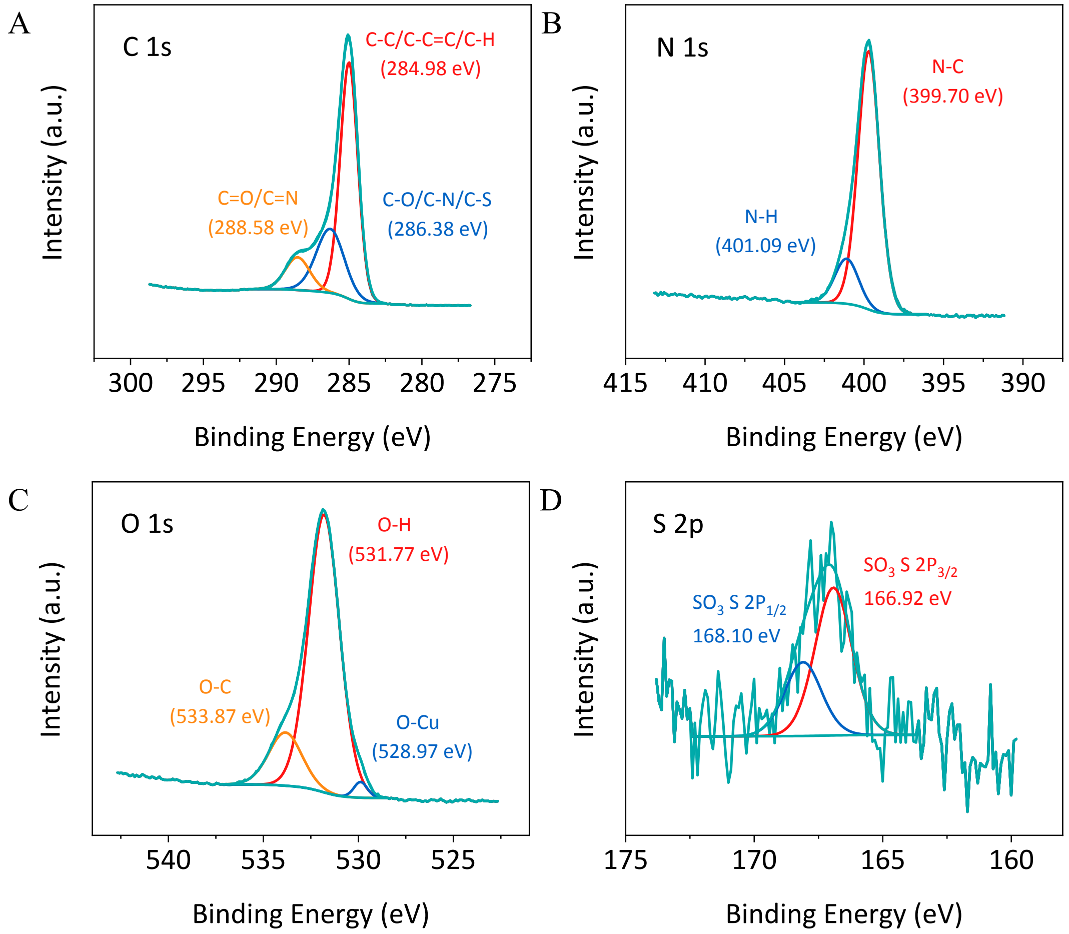


**Figure S18.** Deconvoluted C 1s (**A**), N 1s (**B**), O 1s (**C**) and S 2p (**D**) XPS spectra of UA-imprinted PAn-coated Cu-ZIF NMOFs.

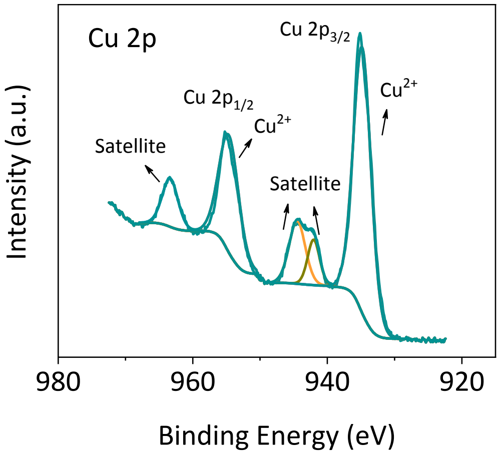


**Figure S19.** Deconvoluted Cu 2p XPS spectra of UA imprinted PAn-coated Cu-ZIF NMOFs treated with $Na_2S_2O_8$.

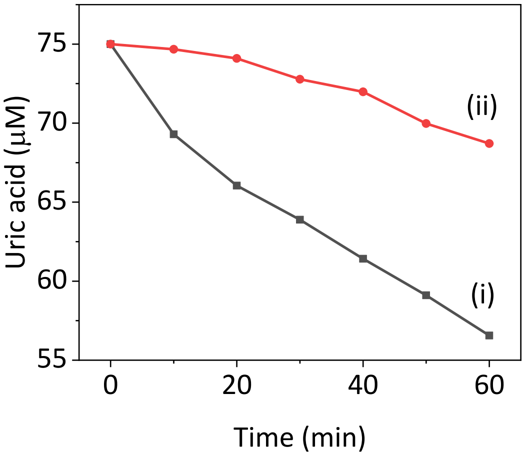


**Figure S20.** Time-dependent concentration changes of UA upon catalytic aerobic oxidation of UA, 75 μM, to allantoin by UA-imprinted PAn-coated Cu-ZIF NMOFs (**i**) and $Na_2S_2O_8$-treated UA-imprinted PAn-coated Cu-ZIF NMOFs (**ii**).

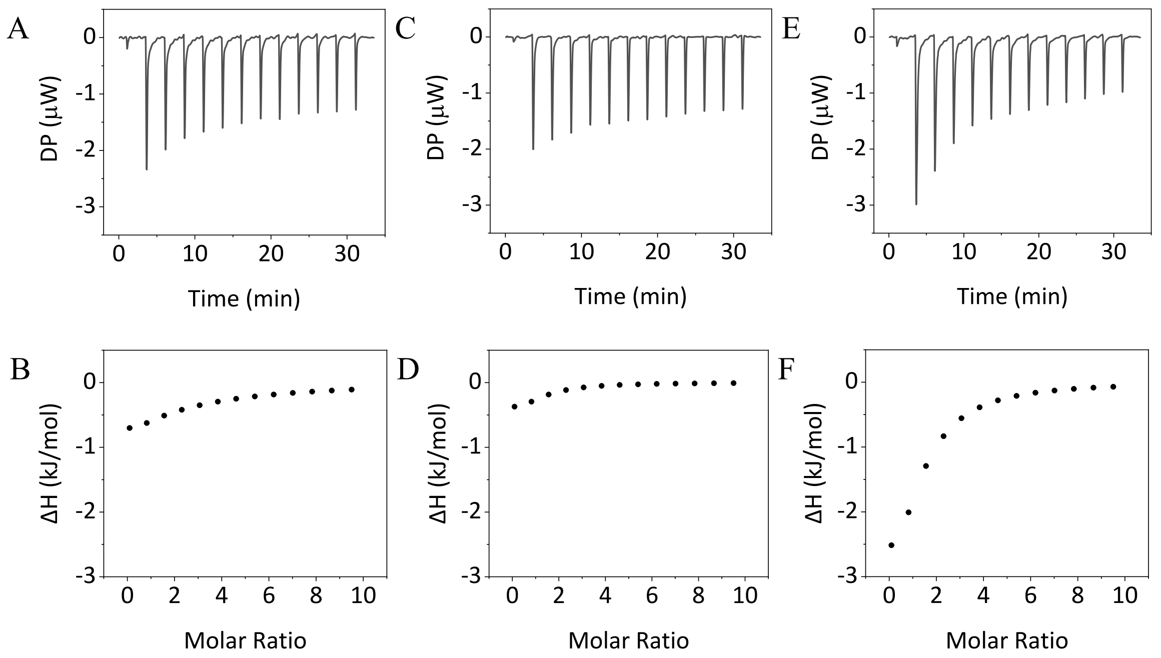


**Figure S21.** The raw calorimetric titration curves (**A**, **C**, **E**) and the integrated curves (**B**, **D**, **F**) generated by injecting UA, 5 mM, into the dispersions of the catalysts, 100 μg $mL^{-1}$: **A**, **B**, Cu-ZIF NMOFs; **C**, **D**, PAn-coated Cu-ZIF NMOFs; **E**, **F**, UA-imprinted PAn-coated Cu-ZIF NMOFs.

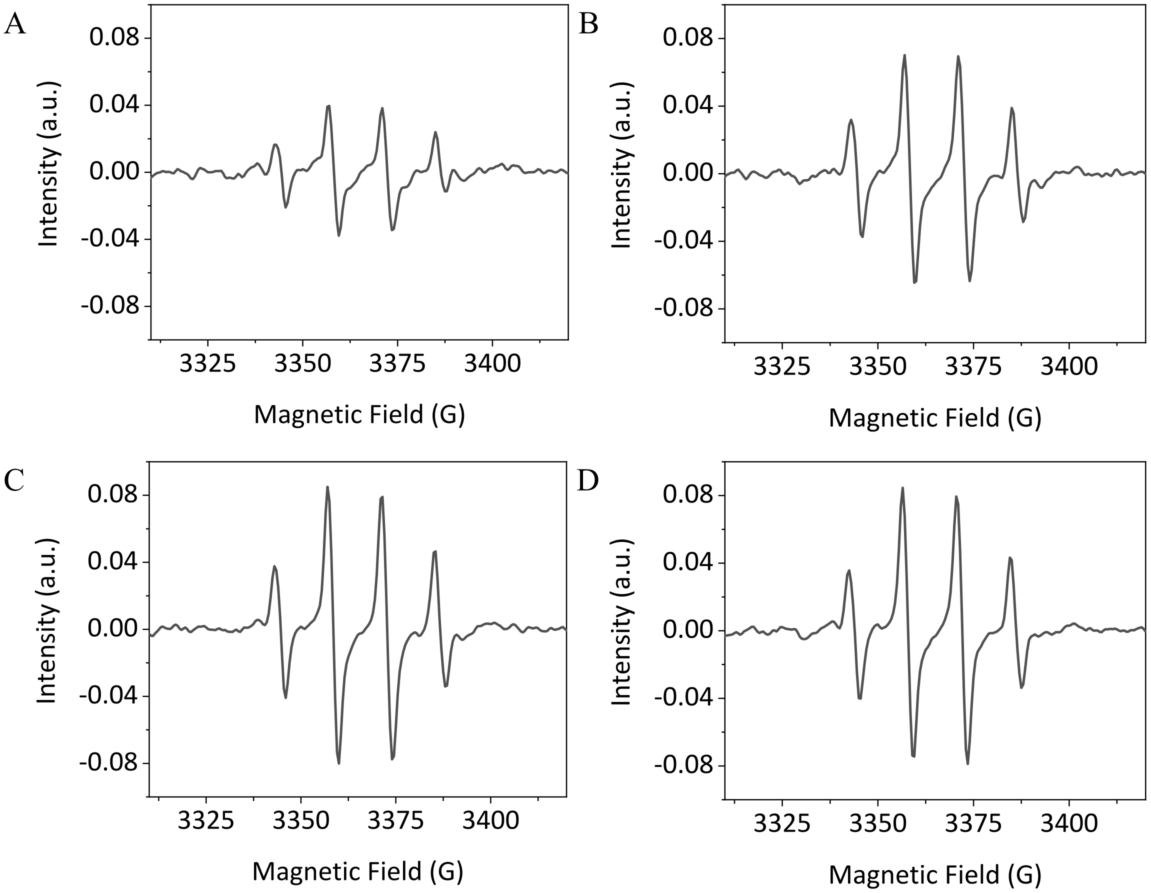


**Figure S22.** EPR spectra generated by the UA-imprinted PAn-coated Cu-ZIF NMOFs in the presence of air, BMPO, 25 mM, and adding variable concentrations of UA: **A**, 25 μM; **B**, 125 μM; **C**, 250 μM; **D**, 500 μM.

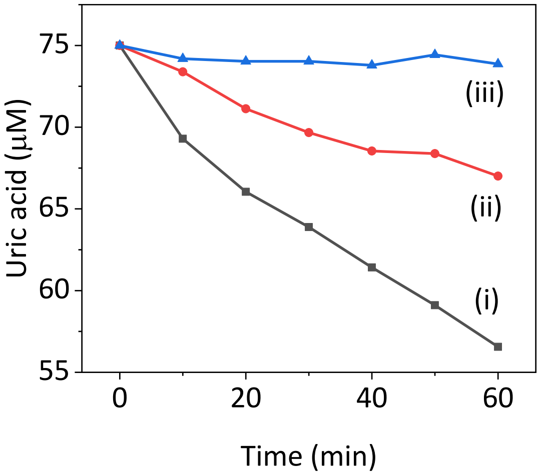


**Figure S23.** Time-dependent concentration changes of UA upon catalyzed aerobic oxidation of UA, 75 μM, to allantoin by UA-imprinted PAn-coated Cu-ZIF NMOFs in the absence (**i**) or presence of different concentration of SOD: **ii**, 0.1 units $L^{-1}$ and **iii**, 0.2 units $L^{-1}$.

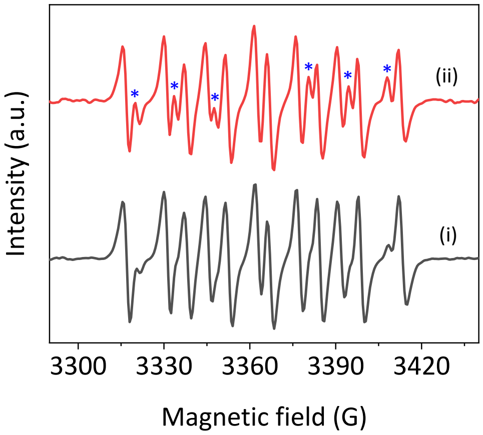


**Figure S24.** Normalized EPR spectra generated by the UA-imprinted PAn-coated Cu-ZIF NMOFs in the presence of air and added DEPMPO, 20 mM (**i**), and by the UA-imprinted PAn-coated Cu-ZIF NMOFs in the presence of air, DEPMPO, 20 mM, and added UA, 0.5 mM (**ii**).

**Identification of the $UA^{+\cdot}$ generated in the mechanistic cycle shown in Figure 5.** An effort to identify the intermediate $UA^{+\cdot}$ in the cycle is presented in **Figure 24**. In this experiment, we employed P-(3,4-dihydro-2-methyl-1-oxido-2H-pyrrol-2-yl)-phosphonic acid, diethyl ester (DEPMPO) as the trapping reagent. **Figure 24**, **curve** (**i**) depicts the EPR spectrum generated by the UA-imprinted PAn-coated Cu-ZIF NMOFs under aerobic condition, in the absence of UA, using DEPMPO as the trapping reagent. The characteristic $O_2^{-\cdot}$ spectrum is observed, consistent with the availability of the polynanozyme hybrid to yield $O_2^{-\cdot}$, even in the absence of UA (due to the presence of $Cu^+$ species in the catalyst, produced within the imprinting process). **Figure 24**, **curve** (**ii**) depicts the EPR spectrum of the imprinted polynanozyme hybrid, in the presence of added UA, under aerobic condition, using DEPMPO as the trapping reagent. Clearly, additional bands, marked with "stars" are observed. These bands are attributed to the intermediate formation of the $UA^{+\cdot}$ (note that these bands are observed only upon addition of UA and are absent upon addition of allantoin). Thus, these EPR results further support the formation of the $UA^{+\cdot}$. (It should be noted that it was impossible to perform the EPR experiment under anaerobic condition since impurities of oxygen and the presence of the $Cu^+$ species in the imprinted polynanozyme generated always a background spectrum of $O_2^{-\cdot}$).

## Effect of UA on $Cu^+$ formation in the imprinted PAn-coated Cu-ZIF NMOFs.

As outlined in the mechanism scheme in **Figure 5**, the formation of $Cu^+$, in the primary step, by oxidation of UA to $UA^{+\cdot}$ is crucial for the oxidase activity of the polynanozyme, that is not present in the bare particles. The efficient electron transfer between $Cu^{2+}$ and UA in the imprinted PAn-coated Cu-ZIF NMOFs was attributed to the concentration of UA in the imprinted sites during the imprinting process (such concentration is not available in the bare particles). The formation of the $Cu^+$ constituent was supported by:

i) Indirect characterization of $UA^{+\cdot}$ by EPR.

ii) XPS quantitative characterization of $Cu^+/Cu^{2+}$ ratio (corresponding to 12.70%/87.30%) upon imprinting the imprinted sites using UA.

iii) XANES measurement.

iv) Enrichment of the $Cu^+$ content in the imprinted PAn-coated Cu-ZIF NMOFs by treatment with UA, 100 μM, under $N_2$ resulting in an $Cu^+/Cu^{2+}$ ratio of 46.16%/53.84%, **Figure S25**.

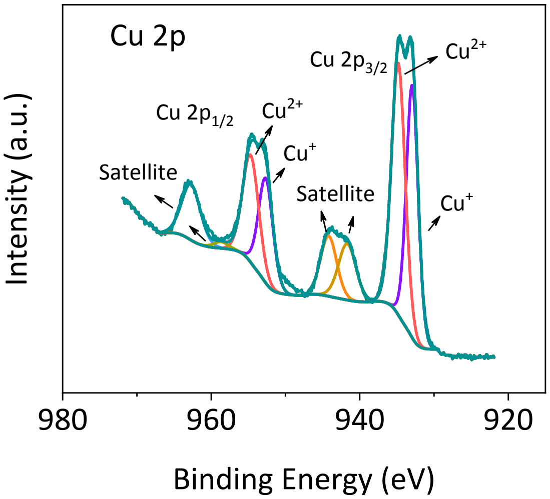


**Figure S25.** Deconvoluted Cu 2p XPS spectra of UA imprinted PAn-coated Cu-ZIF NMOFs treated with UA, 100 μM, under $N_2$.

No such effect and $Cu^+$ formation is observed upon analogue treatment of bare particles with UA under $N_2$.

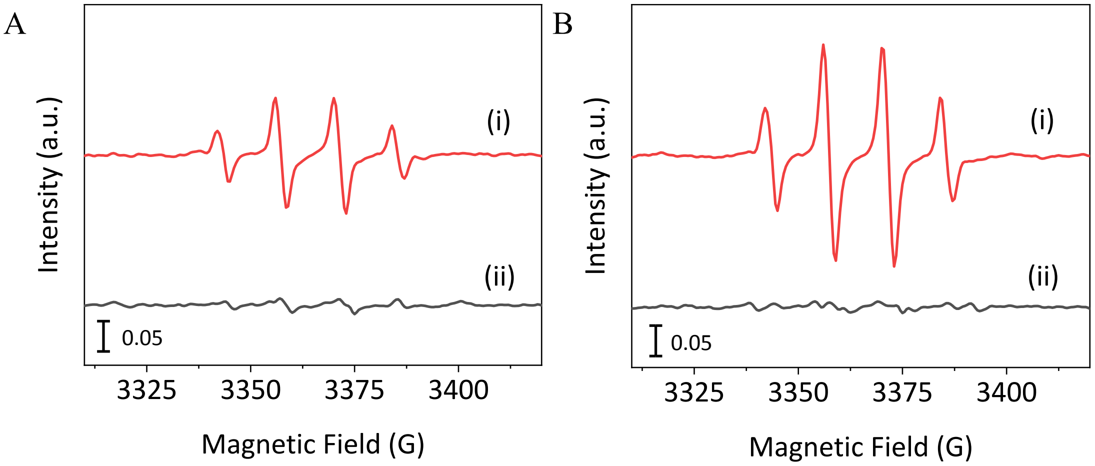


**Figure S26. A**, EPR spectra generated by the Cu-ZIF NMOFs in the presence of $H_2O_2$, 1 mM, and BMPO, 25 mM (**i**), and by the Cu-ZIF NMOFs in the presence of $H_2O_2$, 1 mM, BMPO, 25 mM, and added SOD (**ii**). **B**, EPR spectra generated by the UA-imprinted PAn-coated Cu-ZIF NMOFs in the presence of $H_2O_2$, 1 mM, and BMPO, 25 mM (**i**), and by the UA-imprinted PAn-coated Cu-ZIF NMOFs in the presence of $H_2O_2$, 1 mM, BMPO, 25 mM, and added SOD (**ii**).

**Table S1. Peaks and d-spacing identified in the diffractogram of Cu-ZIF NMOFs**

| Angle (2θ, °) | d-spacing (Å) |
|---|---|
| 14.4264 | 6.1348 |
| 16.9620 | 5.2230 |
| 23.7435 | 3.7444 |
| 29.6738 | 3.0082 |
| 35.5990 | 2.5199 |
| 38.6712 | 2.3265 |
| 44.9999 | 2.0129 |
| 48.8751 | 1.8620 |
| 53.4341 | 1.7134 |
| 58.3351 | 1.5805 |
| 61.5264 | 1.5060 |
| 65.8005 | 1.4181 |
